# A new species of *Illacme* Cook & Loomis, 1928 from Sequoia National Park, California, with a world catalog of the Siphonorhinidae (Diplopoda, Siphonophorida)

**DOI:** 10.3897/zookeys.626.9681

**Published:** 2016-10-20

**Authors:** Paul E. Marek, Jean K. Krejca, William A. Shear

**Affiliations:** 1 Virginia Polytechnic Institute and State University, Department of Entomology, Price Hall, Blacksburg, Virginia, USA; 2 Zara Environmental LLC, 1707 W FM 1626, Manchaca, Texas, USA; 3 Hampden-Sydney College, Department of Biology, Gilmer Hall, Hampden-Sydney, Virginia, USA

**Keywords:** California Floristic Province, paleoendemic, endemic, marble, mesovoid shallow substratum, Kaweah River, foothills, Sierra Nevada forest ecoregion, California interior chaparral and woodlands ecoregion

## Abstract

Members of the family Siphonorhinidae Cook, 1895 are thread-like eyeless millipedes that possess an astounding number of legs, including one individual with 750. Due to their cryptic lifestyle, rarity in natural history collections, and sporadic study over the last century, the family has an unclear phylogenetic placement, and intrafamilial relationships remain unknown. Here we report the discovery of a second species of *Illacme*, a millipede genus notable for possessing the greatest number of legs of any known animal on the planet. *Illacme tobini* **sp. n.** is described from a single male collected in a cave in Sequoia National Park, California, USA. After 90 years since the description of *Illacme*, the species represents a second of the genus in California. Siphonorhinidae now includes *Illacme* Cook & Loomis, 1928 (two species, USA), *Kleruchus* Attems, 1938 (one species, Vietnam), *Nematozonium* Verhoeff, 1939 (one species, South Africa) and *Siphonorhinus* Pocock, 1894 (eight species, India, Indonesia, Madagascar, Vietnam).

## Introduction

The genus *Illacme* is the sole representative of the Siphonorhinidae in the Western Hemisphere. Its closest known relative, *Nematozonium filum* Verhoeff, 1939, is endemic to the Drakensburg Mountains of South Africa (Shelley and Hoffman 2004, Hamer 1998). The current geographical distribution of the Siphonorhinidae in California, Wallacea, Sundaland, Himalayas, Indo-Burma, and southern Africa likely represents remnants of its former range and an ancient radiation predating the breakup of Pangaea more than 200 million years ago (Marek and Bond 2006). There is molecular phylogenetic evidence for monophyly of its order Siphonophorida (Regier et al. 2005, Brewer and Bond 2013, Fernandez et al. 2015). In contrast, it is unclear whether the Siphonorhinidae is a natural group. Because there are so few species in the Siphonorhinidae and little is known about the family from a basic α-taxonomic and a biological perspective, the discovery of a novel species provides significant new data. Here we describe a new species of the genus *Illacme* from a marble cave in Sequoia National Park and provide a world catalog and map of species in the family Siphonorhinidae.

The Siphonorhinidae are members of the subterclass Colobognatha that contains the orders Platydesmida, Polyzoniida, Siphonocryptida, and Siphonophorida (Shear 2011). The Colobognatha are diminutive in size, with most individuals less than 30 mm in length, about a millimeter or less in trunk width, and possessing an oval or circular cross-section. The Platydesmida, Polyzoniida, and Siphonocryptida are typically wider than the Siphonophorida, and are dorsoventrally flattened in segmental cross-section. Some taxa possess elongated paranota further adding to the flattened appearance, and appear platyhelminth-like (e.g., *Brachycybe*, *Hirudicryptus*, *Octoglena*, *Platydesmus*). In contrast, most individuals in the order Siphonophorida are pale thread-like millipedes that are even confused with nematodes by the unaccustomed observer. These millipedes possess the greatest number of leg-bearing trunk segments of any animal. One female specimen of *Illacme plenipes* Cook & Loomis, 1928 possesses a superlative 192 diplosegments. The trunk rings are translucent and lightly pigmented and lack the heavy cuticle that many chilognathan diplopods possess. Despite the delicate nature of their exoskeleton, siphonophoridans have a variety of cuticular adornments—including spines, tubercles, and silk-secreting setae (Read and Enghoff 2009, Marek et al. 2012).

The rings of colobognath millipedes are uniform in appearance throughout the length of the trunk, except those of some Platydesmida (genus *Andrognathus*) with anteriorly projecting ozopores on the fifth ring and Platydesmida and Siphonocryptida with color patterns that vary antero-posteriorly (Shear and Marek 2009, Enghoff 2011). The cephalic morphology of colobognaths is generally regarded as highly derived relative to other Diplopoda, making it difficult to draw homology to known structures (Enghoff et al. 2015). The orders Siphonocryptida and Polyzoniida possess a cluster of simple eyes; in Platydesmida and Siphonophorida eyes are absent. However, a few platydesmidan taxa possess pigmented patches on the cuticle where the eyes would normally occur. The Colobognatha (meaning “abbreviated jaw”) are characterized by reduced mouthparts. Their heads are often small in relation to the trunk and appear triangular in anterior view, while other millipedes have larger, subspherical heads supporting musculature required for strong chewing action by the mandibles. This modification reaches a pinnacle in the Siphonophoridae, which have heads drawn out into long beaks, and mandibles that are highly simplified and styliform, with gnathochilarial components fused and reduced. The Siphonorhinidae possess mouthparts somewhere in between with components that remain identifiable and able to be homologized with those of other non-colobognath millipedes.

In contrast with their derived cephalic morphology, the order Siphonophorida possesses a primitive trunk architecture for helminthomorph diplopods, including unfused rings composed of a free sternite, pleurite, and tergite. The siphonorhinid mouthparts are presumed to be ancestral to the highly derived siphonophorid beak, and based on these features, siphonorhinids are hypothesized to be a basal sister group to the remaining siphonophoridan taxa. Based on molecular phylogenetics, the Siphonophorida are sister to a clade formed by exemplars of the Polyzoniida and Platydesmida (Regier et al. 2005, Sierwald and Bond 2007). However, the family Siphonorhinidae has yet to be sampled by recent phylogenomic estimations of the class Diplopoda (Brewer and Bond 2013, Fernandez et al. 2015).

*Illacme* species and their colobognathan relatives exhibit true anamorphosis (euanamorphosis), whereby six-legged hatchlings develop into adulthood in coordination with the addition of new segments (Enghoff et al. 1993). The addition of new segments lengthens the body and adds legs, which develop shortly after segment formation. This process continues beyond attainment of sexual maturity for an indeterminate amount of time, and imparts high variability and a very large number of segments in euanamorphic taxa. A paratype female of *Illacme plenipes* collected by O.F. Cook in 1926 possesses 192 segments and 750 legs (Cook and Loomis 1928). The age of this exceptionally segmented individual is unknown, but likely to be several years. While diplosegmentation and sequentially repeated leg-bearing segments serve to provide force for burrowing, the superlative segment count in *Illacme plenipes* seems unwarranted and perhaps serves another function. The role is unclear, and several hypotheses have been suggested including: burrowing in deeper soil, clinging to rocks, or lengthening the gut to digest low nutrient food (Marek et al. 2012). As in the highly elongate geophilomorph centipedes, the long, flexible body may be an adaptation to negotiating narrow, pre-existing spaces in the soil.

In the western U.S. (Arizona, California, Texas), Siphonophorida occur in moist refugia within more arid habitats. However, many tropical siphonophoridans occur in mesic habitats and in rainforests that are continuously wet. The microhabitats of siphonophoridan species are usually within deep substrata and individuals are frequently discovered beneath large stones (e.g., *Illacme plenipes* in California) and embedded inside large decaying logs (e.g., *Siphonophora* species in Central America). Persistence in these microhabitats is consistent with their morphology, including a lack of eyes, depigmented exoskeleton, shortened legs, and an elongate flexible body. The Siphonophorida in Arizona and California are found in relatively mesic oak woodlands in mountain foothills, including those of the Coast Ranges (CA), Sierra Nevada (CA), and Madrean Sky Islands (AZ).

## Methods and results

From 2002 to 2004, scientists led formal biological surveys of caves in Sequoia and Kings Canyon National Parks for invertebrates, including arachnids, myriapods, and hexapods. From 2006 to 2009 several follow up visits yielded incidental collections, and among these specimens was one sample of *Illacme tobini* sp. n. from Lange Cave, collected by JKK on 9 October 2006. Three years after this discovery, myriapod specialists made three additional expeditions to Lange Cave and surrounding habitats to search for additional material for description of the species. Collecting effort was focused within the cave, and surface searches around the cave entrance were carried out. More intensive searches were conducted at the the confluence of Cave, Yucca, and Cascade creeks—the general area where *Illacme tobini* sp. n. was discovered. During field expeditions by PEM from 2010–2012, 63 additional localities in the foothills of the Sierra Nevada from El Dorado National Forest southward to the Tehachapi Mountains were explored for *Illacme tobini* sp. n. Using techniques previously developed for *Illacme plenipes* (and applied to *Illacme tobini* sp. n.), the undersides of large stones were examined. The bases of decaying logs and leaf litter were also searched, albeit an improbable microhabitat since previous collections of U.S. siphonophoridans were rarely encountered in these areas. Live millipedes were collected by hand, or in some cases lifted with a paintbrush or forceps if necessary. In spite of all of this additional effort, field biologists found no additional specimens.

**Figure 1.**
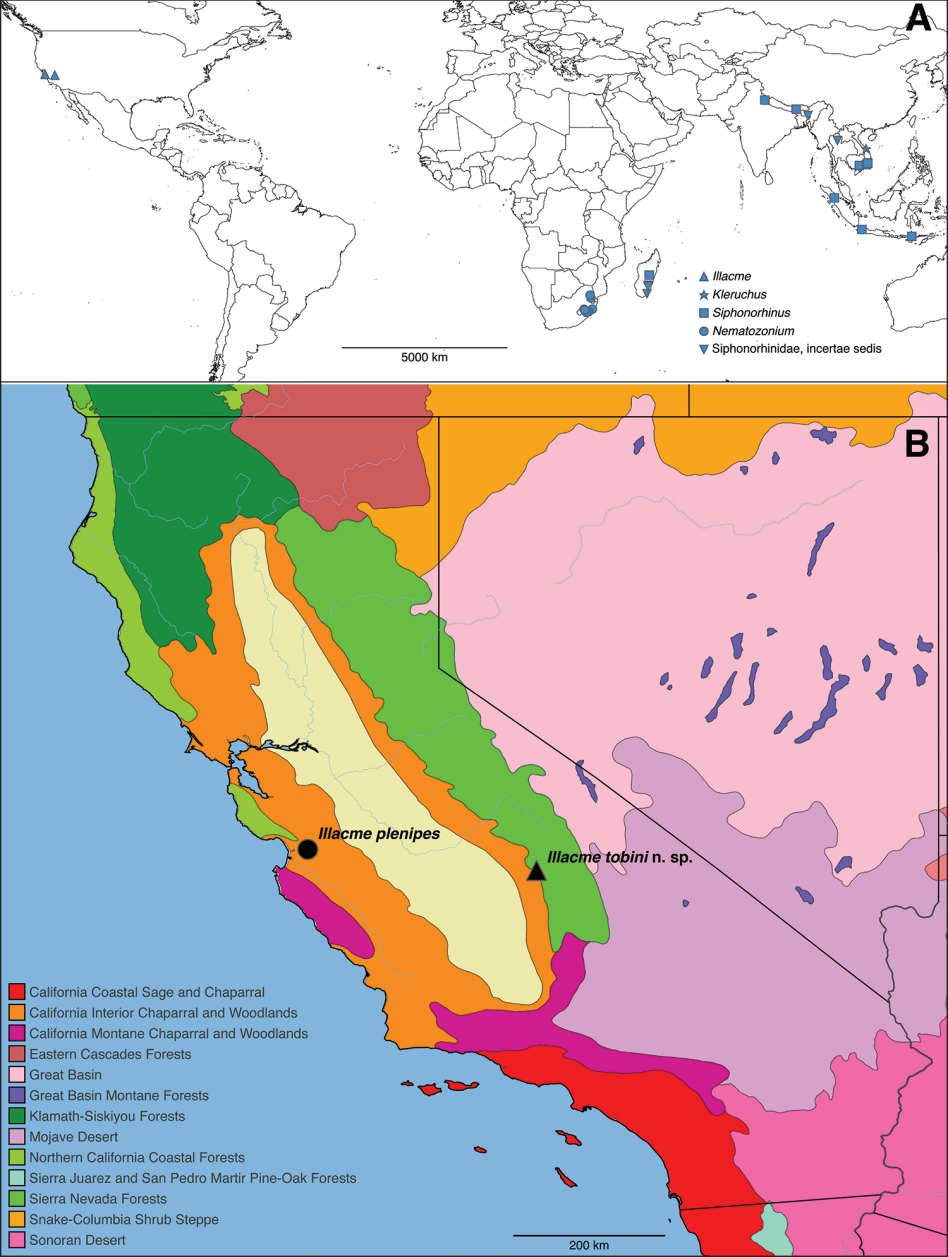
**A** Distribution of the millipede family Siphonorhinidae, and **B** of the genus *Illacme*. Terrestrial ecoregions according Olson et al. (2001).

Lange Cave is 240 km east of its congener *Illacme plenipes* that occurs in San Benito County California (Fig. 1). The single live specimen was sacrificed and preserved in 80% ethanol. Forty segments from the midbody were removed and preserved in 100% ethanol and archived at -20 °C nine years later in an attempt to preserve DNA. The remnants of the holotype (anterior and posterior sections) were removed, dried at room temperature, and mounted on a standard SEM pin stub mount (Ø12.7 mm × 8 mm pin height) with double sided carbon conductive tape. Specimens were coated with 10 nm of platinum and palladium metals with a Leica EM ACE600 high vacuum coater (Wetzlar, Germany), and stored with silica gel desiccant until ready for examination.

Genomic DNA was extracted and purified from half of the ethanol-preserved tissue from the midbody section using a Qiagen DNeasy tissue extraction protocol. The remaining half of the tissues have been retained in the VTEC frozen tissue collection. Standard kit protocol was followed and the DNA was eluted from the spin column with one round of 50 µL AE buffer. Genomic DNA was archived at -20 °C in the freezer collections at the VTEC. A segment of the cytochrome c oxidase I gene (COI), was amplified using polymerase chain reaction and the thermal cycling steps of Hebert et al. (2003) and with the universal DNA barcoding primers LCO1490 and HCO2198 of Folmer et al. (1994). Polymerase chain reaction of the COI barcoding region of the single *Illacme tobini* sp. n. individual and visualization of amplifications on a 12% agarose gel, did not indicate the presence of DNA based on comparison with the negative and positive controls. A second PCR was repeated with the amplification product from the first reaction, in case a low concentration of DNA from *Illacme tobini* sp. n. was present. The second reaction showed the same results and a lack of DNA based on comparison with the controls.

### Descriptive taxonomy

**Figure 2.**
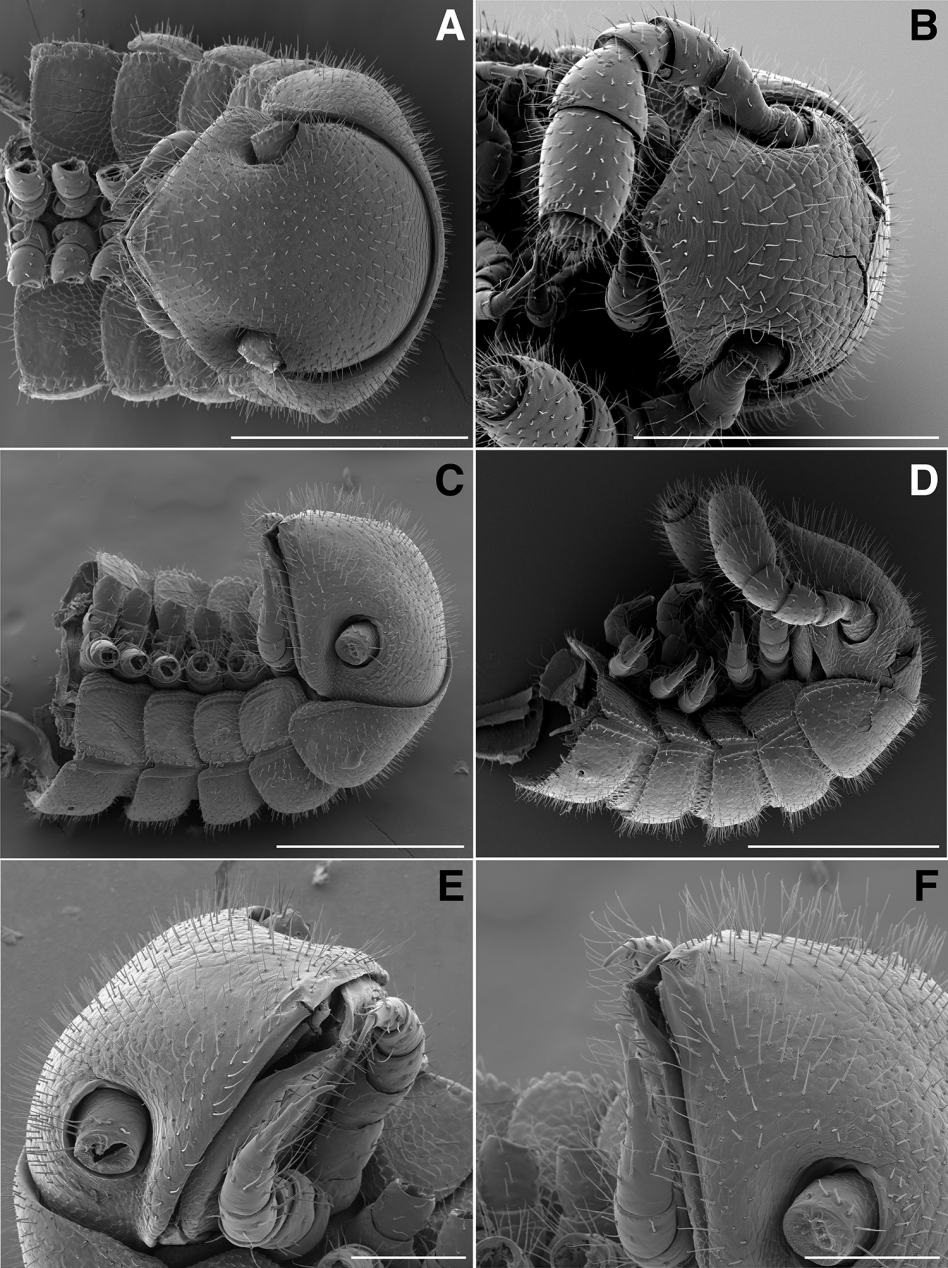
**A** Dorsal view of head, antennae and rings 1–5 of *Illacme tobini* sp. n. (scale bar 300 µm) **B** the same of *Illacme plenipes* (scale bar 300 µm) **C** Lateral (right) view of head and rings 1–5 of *Illacme tobini* sp. n. (scale bar 300 µm) **D** the same of *Illacme plenipes* (scale bar 300 µm). *Illacme tobini* sp. n.: **E** anterolateral (right) view of head and first leg pair (scale bar 100 µm) **F** lateral (left) view of head and first leg pair, antennae broken off at base (scale bar 100 µm). (Catalog #s: *Illacme tobini* sp. n. MPE00735, *Illacme plenipes* SPC000932.)

**Figure 3.**
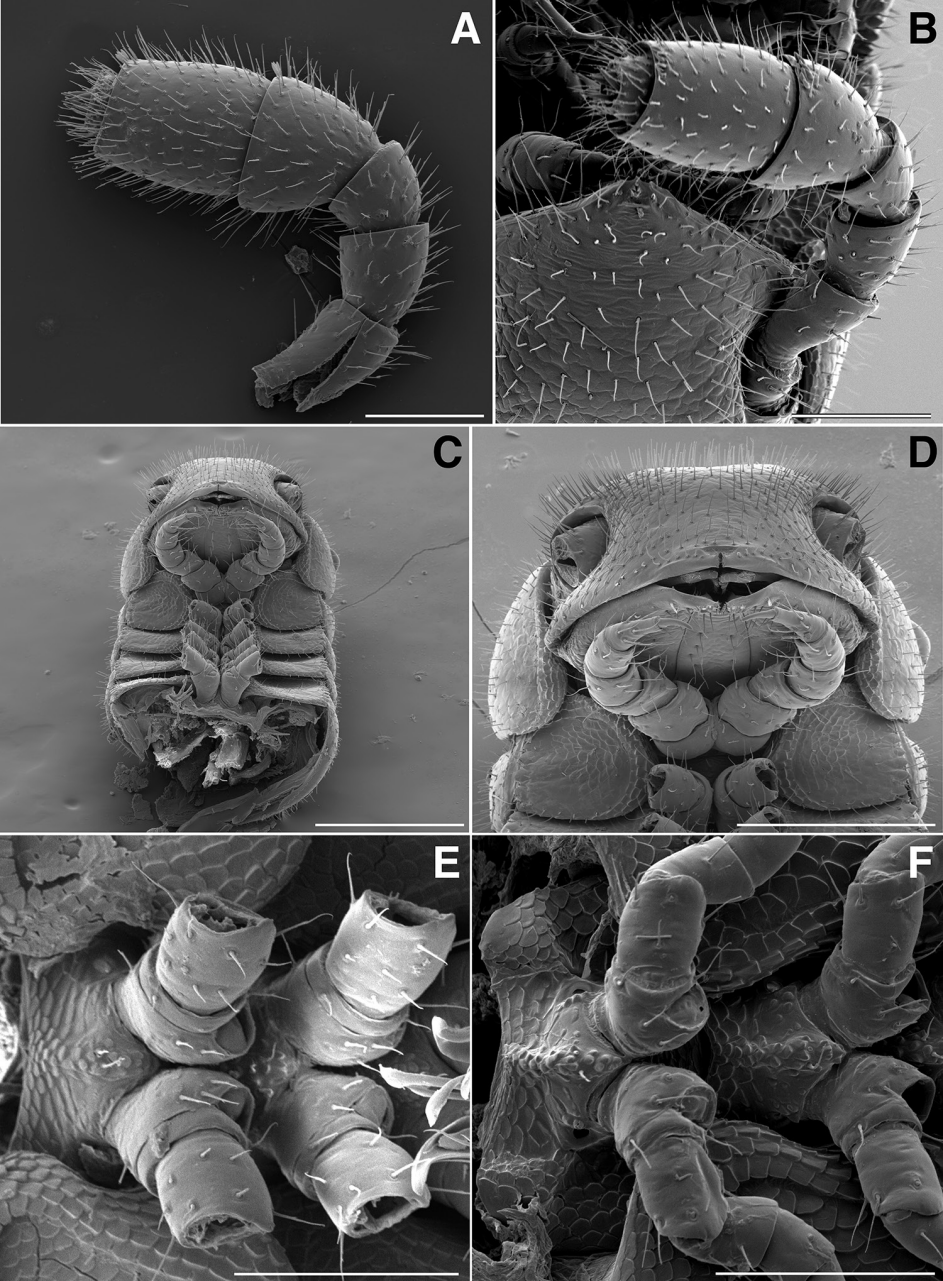
**A** Lateral (right) view of antenna of *Illacme tobini* sp. n. (scale bar 100 µm) **B** the same of *Illacme plenipes* (scale bar 100 µm). *Illacme tobini* sp. n.: **C** ventral view of head and rings 1–5 (scale bar 300 µm) **D** the same of head and rings 1–3, magnified view (leg pairs 2–6 broken off at prefemur-femur joint) (scale bar 200 µm) **E** Ventral view of rings 6 and 7 with sternites, pleurites and leg bases of *Illacme tobini* sp. n. (scale bar 100 µm) **F** the same of *Illacme plenipes* (scale bar 100 µm). (Catalog #s: *Illacme tobini* sp. n. MPE00735, *Illacme plenipes* SPC000932.)

**Figure 4.**
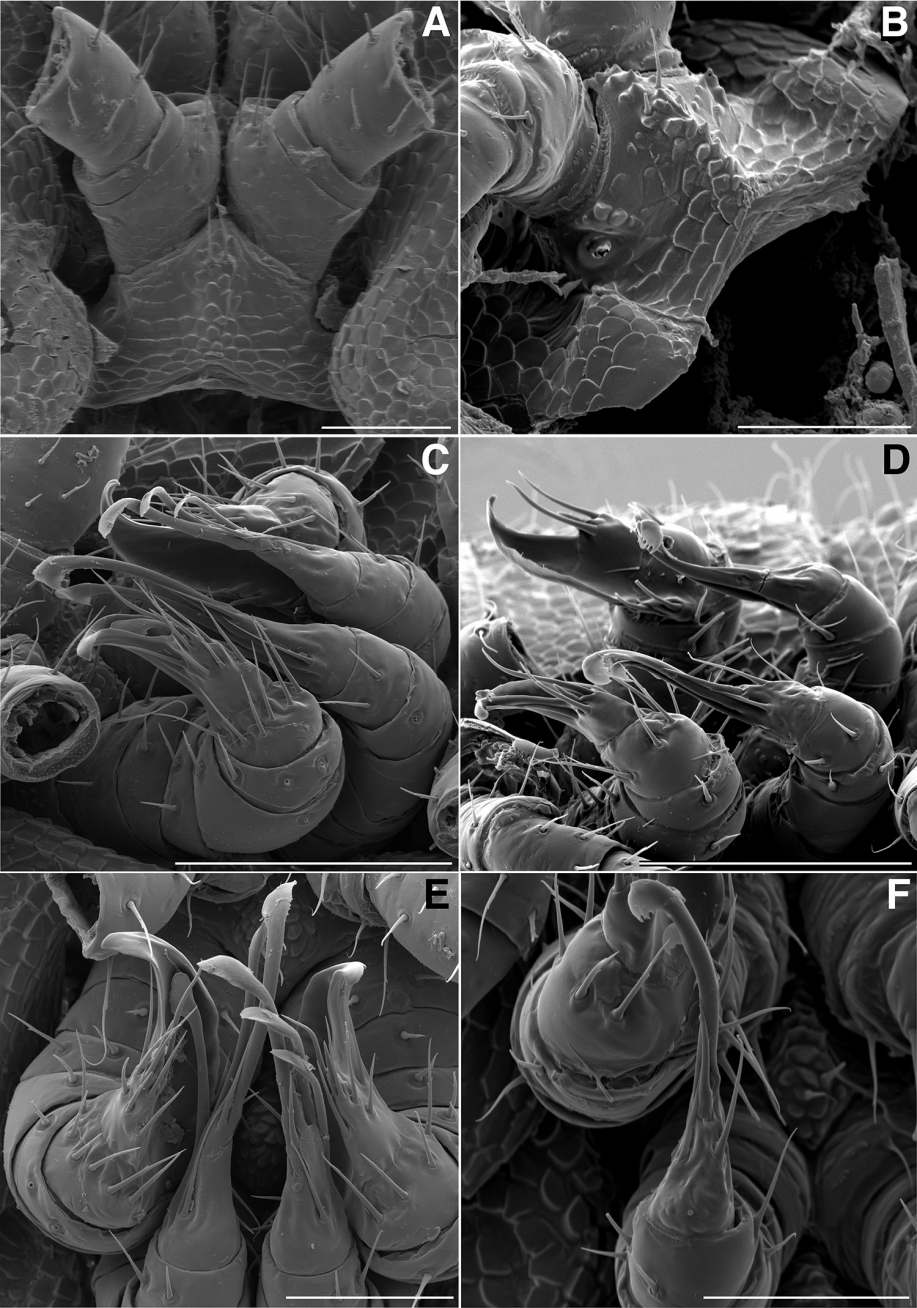
**A** Anterior view of ring 6 sterna of *Illacme tobini* n. sp (scale bar 50 µm) **B** anterolateral (left) view of the same of *Illacme plenipes* (scale bar 50 µm) **C** Lateral (right) view of gonopods (leg pairs 9 and 10) of *Illacme tobini* sp. n. (scale bar 100 µm) **D** the same of *Illacme plenipes* (scale bar 100 µm) **E** Ventral view of gonopods of *Illacme tobini* sp. n. (centered on right posterior gonopod, leg-pair 10) (scale bar 50 µm) **F** the same of *Illacme plenipes* (scale bar 50 µm). (Catalog #s: *Illacme tobini* sp. n. MPE00735, *Illacme plenipes* SPC000932.)

**Figure 5.**
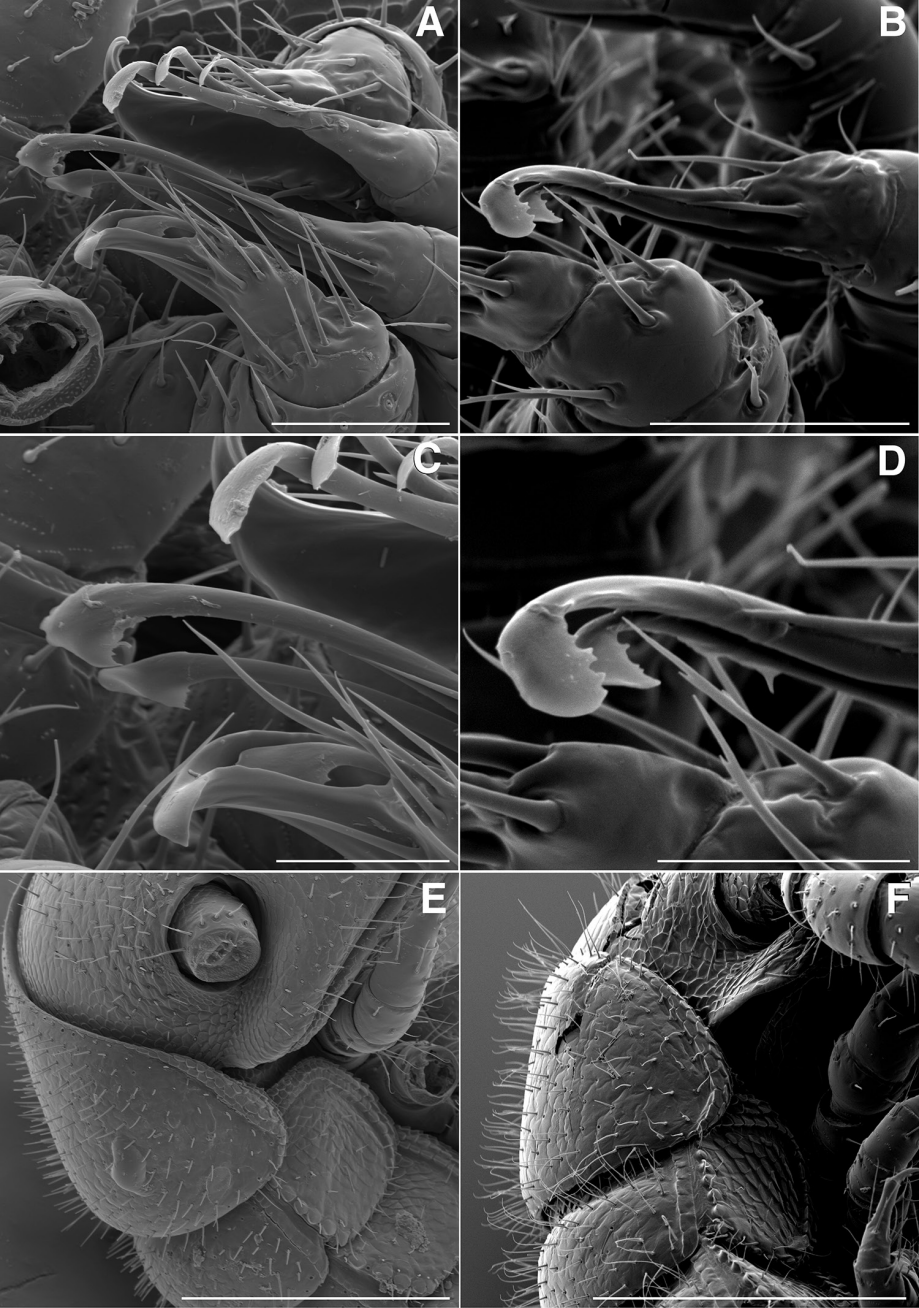
**A** Lateral (right) view of gonopods of *Illacme tobini* sp. n. (centered on right posterior gonopod) (scale bar 50 µm) **B** the same of *Illacme plenipes* (scale bar 50 µm) **C** Lateral (right) view of right posterior gonopod apex of *Illacme tobini* sp. n. (scale bar 25 µm) **D** the same of *Illacme plenipes* (scale bar 25 µm) **E** Lateral (right) view of head, collum, and rings 2, 3 of *Illacme tobini* sp. n. (scale bar 200 µm) **F** the same of *Illacme plenipes* (scale bar 200 µm). (Catalog #s: *Illacme tobini* sp. n. MPE00735, *Illacme plenipes* SPC000932.)

**Figure 6.**
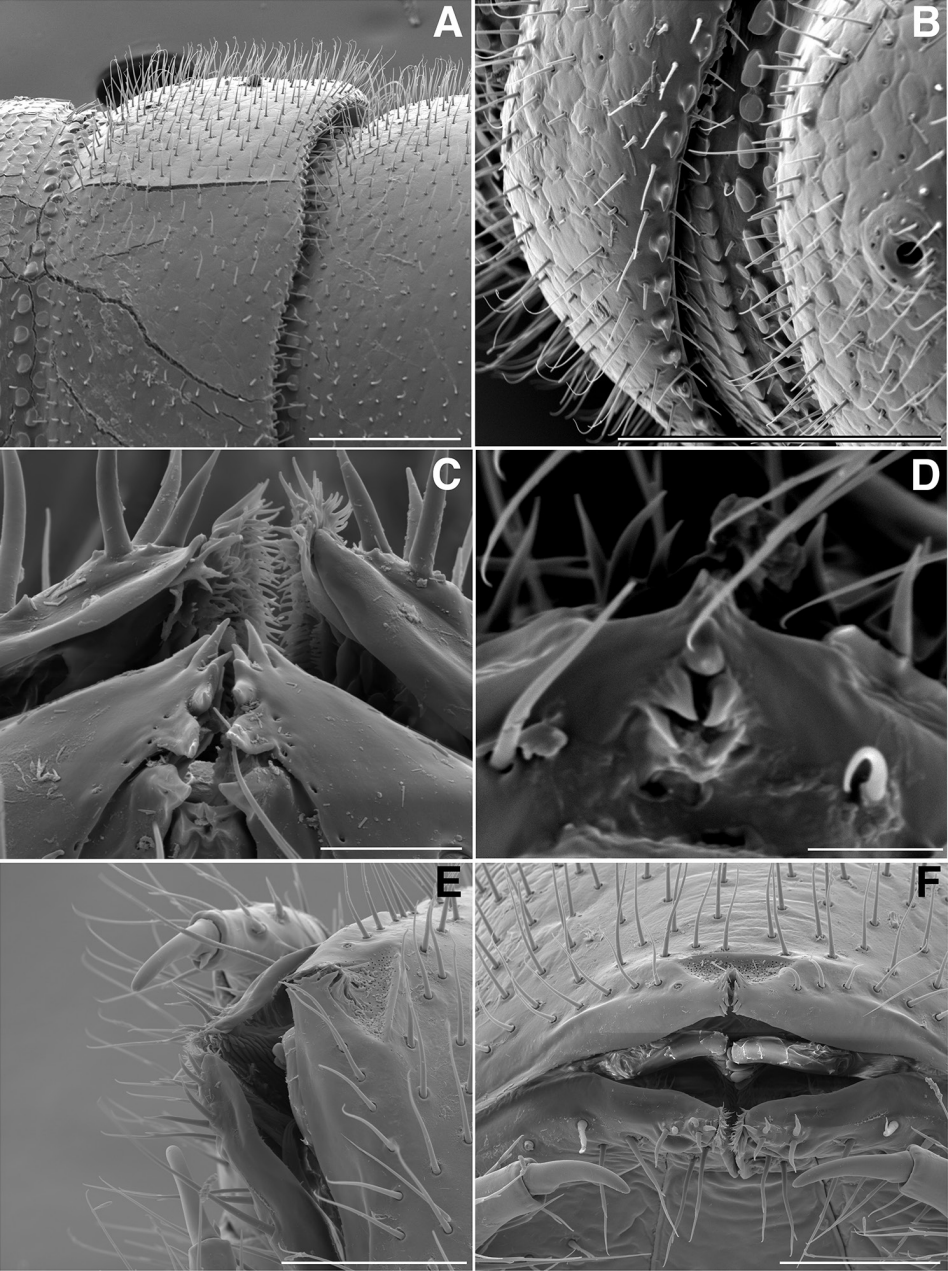
**A** Dorsolateral (left) view of tenth prozonite and metatergite of *Illacme tobini* sp. n. (scale bar 100 µm) **B** the same of *Illacme plenipes* (scale bar 100 µm) **C** Dorsal view of anterior region of head and labrum of *Illacme tobini* sp. n. (scale bar 10 µm) **D** the same of *Illacme plenipes* (the gnathochilarial apices can be seen projecting beneath the medially split labrum) (scale bar 10 µm). *Illacme tobini* sp. n.: **E** anterolateral (left) view of head and first leg pair (scale bar 50 µm) **F** anterior view of head with gnathochilarium open showing flabellate mandibles (scale bar 50 µm). (Catalog #s: *Illacme tobini* sp. n. MPE00735, *Illacme plenipes* SPC000932.)

For comparison with *Illacme tobini* sp. n., we examined the 17 known specimens of *Illacme plenipes* from the Smithsonian Institution (USNM), Florida State Collection of Arthropods (FSCA), Virginia Museum of Natural History (VMNH), and Virginia Tech Insect Collection (VTEC). The *Illacme tobini* sp. n. specimen label information was databased in Symbiota Collections of Arthropods Network (http://symbiota4.acis.ufl.edu/scan/portal/). Due to the sensitivity of its cave habitat, locality details are withheld publicly on SCAN, and are available upon request from the authors. The following dimensions were measured for *Illacme plenipes* and *Illacme tobini* sp. n.: (1) body length: from anterior margin of labrum to posterior margin of paraprocts, BL; (2) head width, HW; (3) head length, HL; (4) interantennal socket width, ISW; (5) antennomere 6 width, AW; (6) collum width, CW; (7) metazonite width at 1/4 length of body, W1; (8) metazonite length at 1/4 length of body, L1; (9) metazonite height at 1/4 length of body, H1; (10) first apodous metazonite width, AS1; (11) anterior gonopod article 7 width, A7W; and (12) posterior gonopod article 7 width, P7W. The 12 measurements refer to 1–10, 17 and 18 used in Marek et al. (2012). Specimens were measured from digital scanning electron and light micrographs using the segmented line measurement tool in ImageJ64 (Rasband 2011). Measurements are recorded in millimeters and this unit is hereafter excluded throughout the paper. The number of segments were counted and legs calculated using the formula l = ((p + a) × 4)–(a × 4)–(10), where l is the number of legs, p is the number of podous tergites (each bearing four legs), a is the number of apodous tergites (without legs), and 10 is the number to be subtracted because the first tergite (the collum) is legless and second through fourth tergites (the millipede thorax) each have two legs. Examination of specimens were accomplished with a Leica M125 stereomicroscope with eyepiece reticules (Wetzlar, Germany). Scanning electron micrographs were taken of palladium/platinum coated structures with an FEI Quanta 600 FEG environmental SEM (Hillsboro, Oregon). The figures are of male specimens, unless otherwise indicated, and Figs 2–6, 8–11, 15–17, 18, and 19 show *Illacme tobini* sp. n. and *Illacme plenipes* (VTEC catalog # SPC000932) side by side for comparison. The identification and terminology of antennal sensilla followed that of Nguyen Duy-Jacquemin (1974) and Chung and Moon (2006). Terminology of mouthparts is from Silvestri (1903) and Koch (2015). Museum abbreviations follow Marek et al. (2014), and supplemental abbreviations are the following: HT = holotype; PT = paratype; LT = lectotype; ST = syntype; nec = but not; and sic = misspelling. The National Geospatial-Intelligence Agency GEOnet Names Server (NGA GNS) was used to query names of historical type localities for their geographical coordinates, using the “include historical records” option (http://geonames.nga.mil/gns/html/). The elevation of the type locality was determined from a U.S. Geological Survey quadrangle topographic map: Giant Forest Quadrangle, 7.5-minute series (USGS 2015). The uncompressed and uncropped scanning electron micrographs of *Illacme tobini* sp. n. are archived in the Dryad Data Repository at https://doi.org/10.5061/dryad.tk0b8 under a public domain CC0 Creative Commons license.

**Figure 7.**
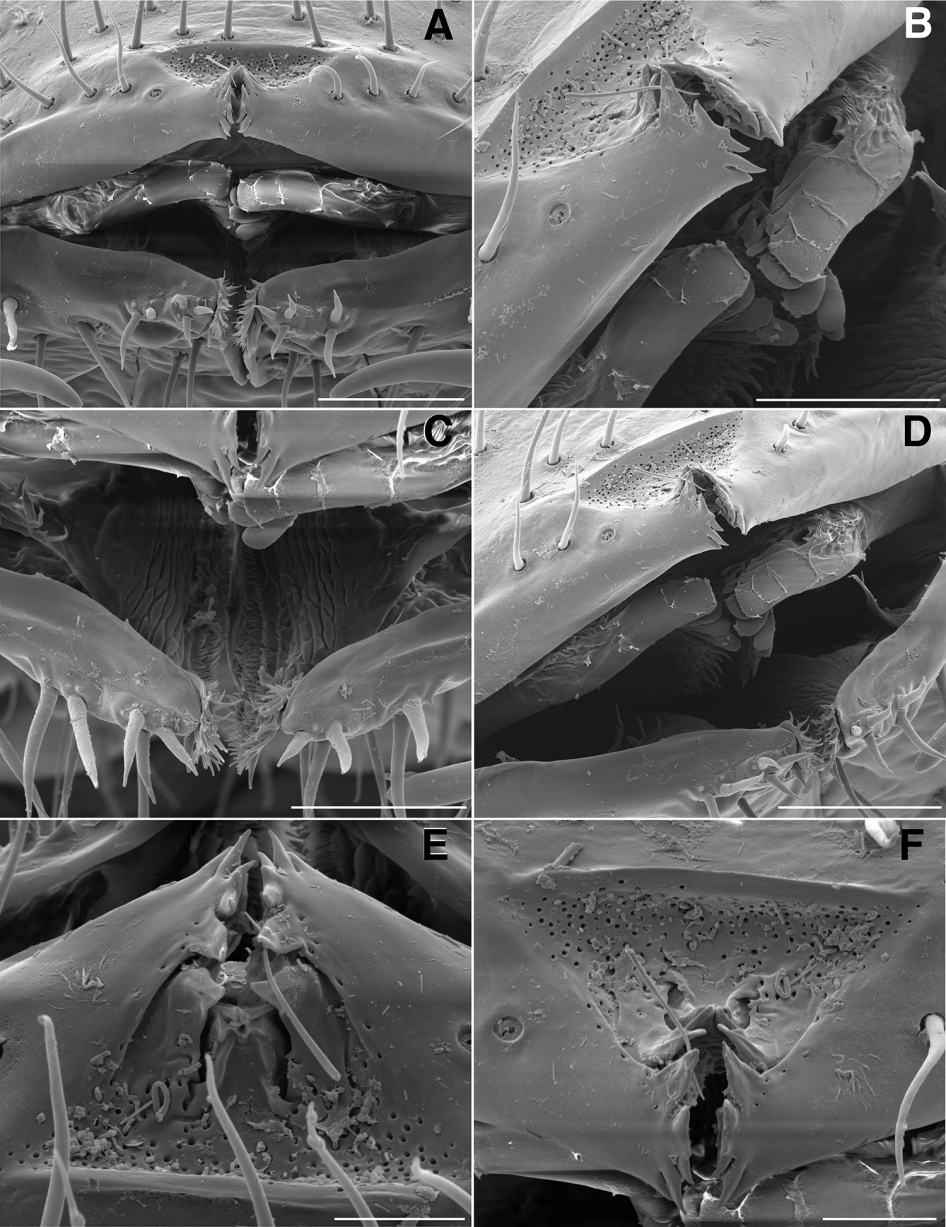
*Illacme tobini* sp. n.: **A** anterior view of head with gnathochilarium open showing flabellate mandibles (scale bar 30 µm) **B** anterolateral (right) view of head with gnathochilarium open showing mandibles and pectinate lamella with numerous rows of jagged ventrally projecting serrulae (scale bar 20 µm) **C** dorsal view of V-shaped endochilarial frontal body with fringed lobes (spatulae) protruding through gnathochilarial stipes (scale bar 20 µm) **D** anterolateral (right) view of open mouth and keel-shaped pectinate lamella of the mandible nested in V-shaped groove of the endochilarium (scale bar 30 µm) **E** dorsal view of labrum with deep medial incision (scale bar 10 µm) **F** anterior view of labrum with heavily porous surface (scale bar 10 µm). (Catalog #: *Illacme tobini* sp. n. MPE00735.)

**Figure 8.**
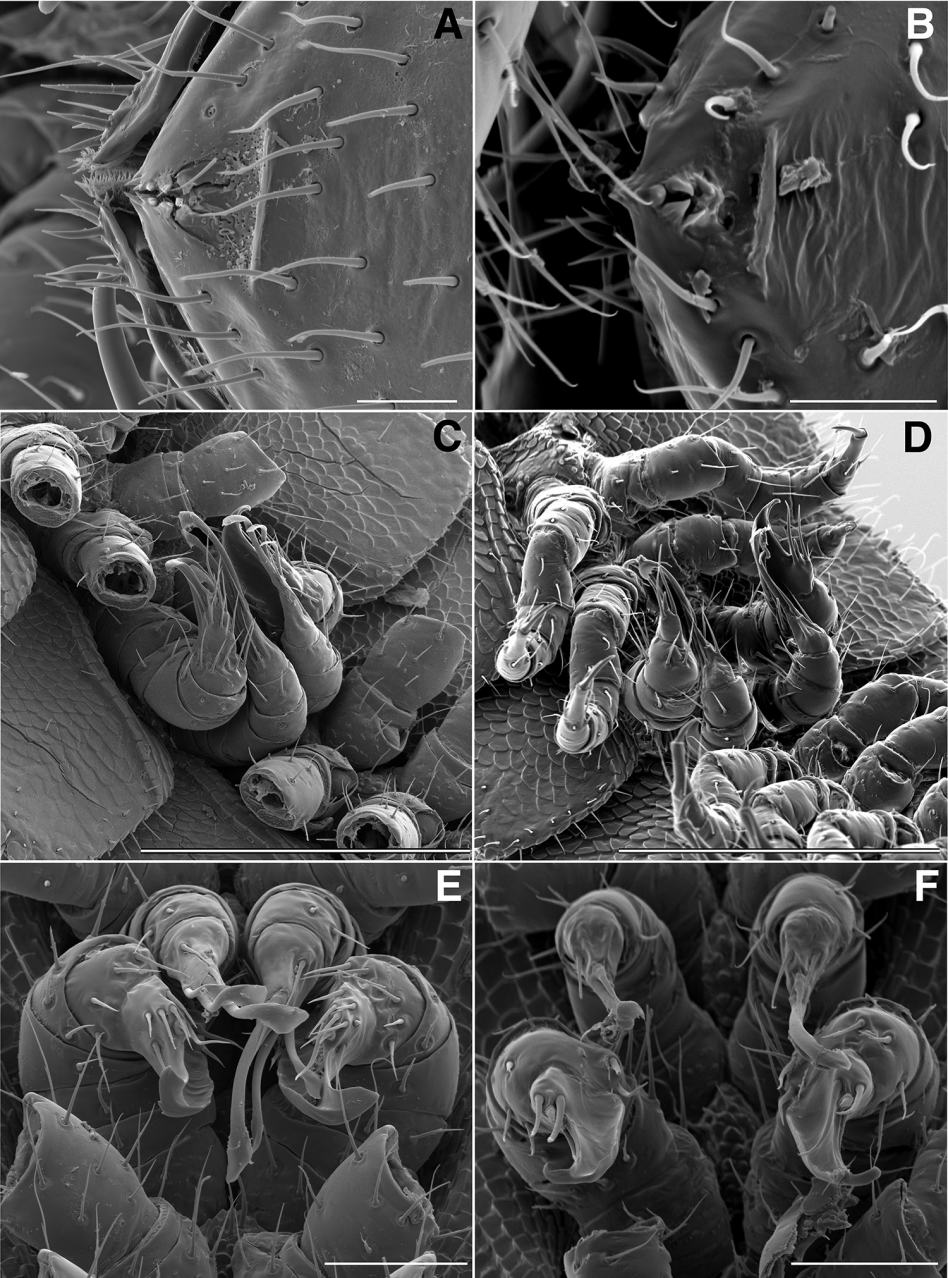
**A** Dorsal view of anterior region of head and labrum of *Illacme tobini* sp. n. (scale bar 20 µm) **B** the same of *Illacme plenipes* (scale bar 20 µm) **C** Ventrolateral (right) view of gonopods of *Illacme tobini* sp. n. (scale bar 200 µm) **D** the same of *Illacme plenipes* (scale bar 200 µm) **E** Anteroventral view of gonopods of *Illacme tobini* sp. n. (scale bar 50 µm) **F** the same of *Illacme plenipes* (scale bar 50 µm). (Catalog #s: *Illacme tobini* sp. n. MPE00735, *Illacme plenipes* SPC000932.)

**Figure 9.**
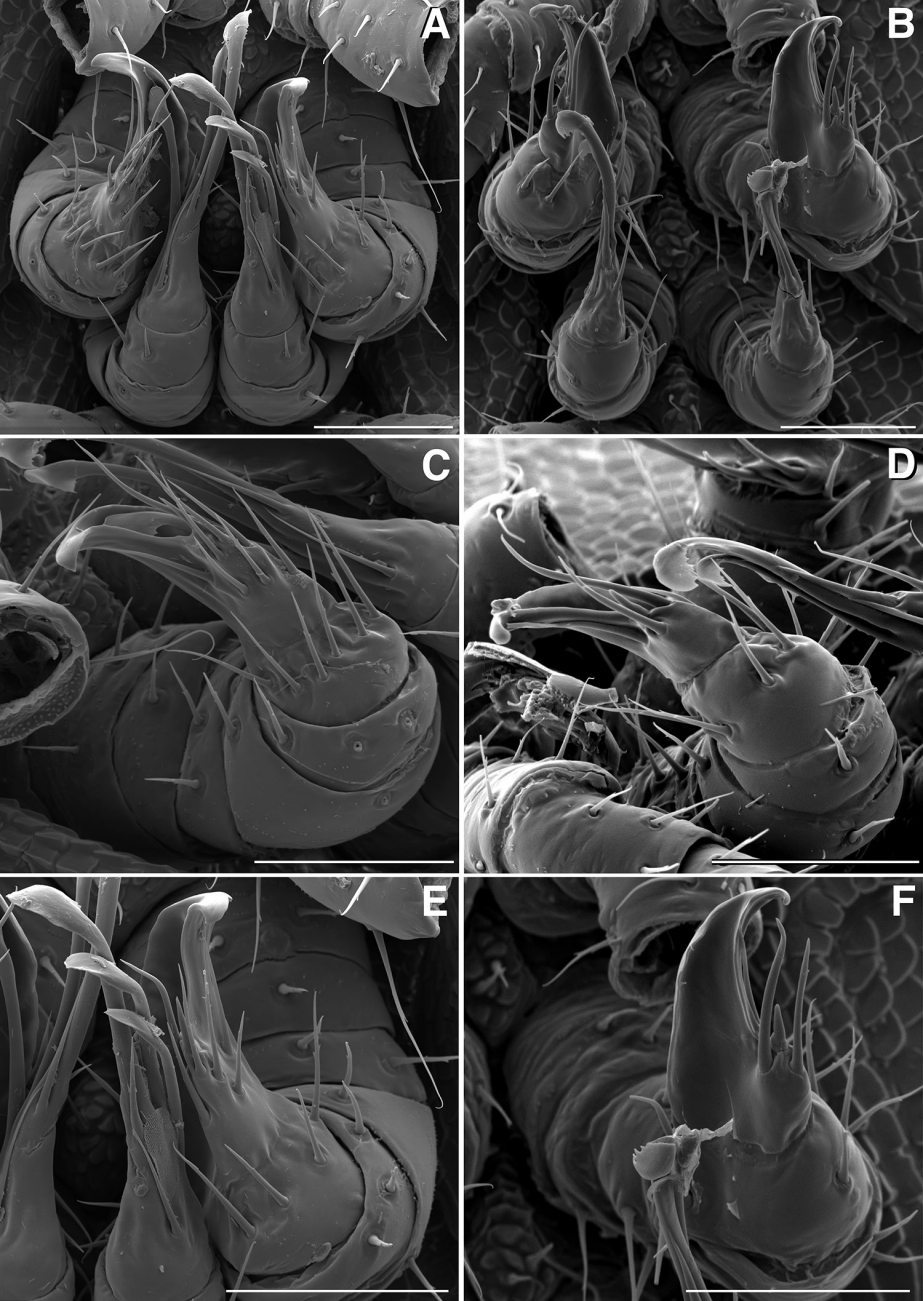
**A** Ventral view of gonopods of *Illacme tobini* sp. n. (scale bar 50 µm) **B** the same of *Illacme plenipes* (scale bar 50 µm) **C** Lateral (right) view of right anterior gonopod (leg-pair 9) of *Illacme tobini* sp. n. (scale bar 50 µm) **D** the same of *Illacme plenipes* (scale bar 50 µm) **C** Ventral view of gonopods of *Illacme tobini* sp. n. (centered on right anterior gonopod) (scale bar 50 µm) **D** the same of *Illacme plenipes* (scale bar 50 µm). (Catalog #s: *Illacme tobini* sp. n. MPE00735, *Illacme plenipes* SPC000932.)

**Figure 10.**
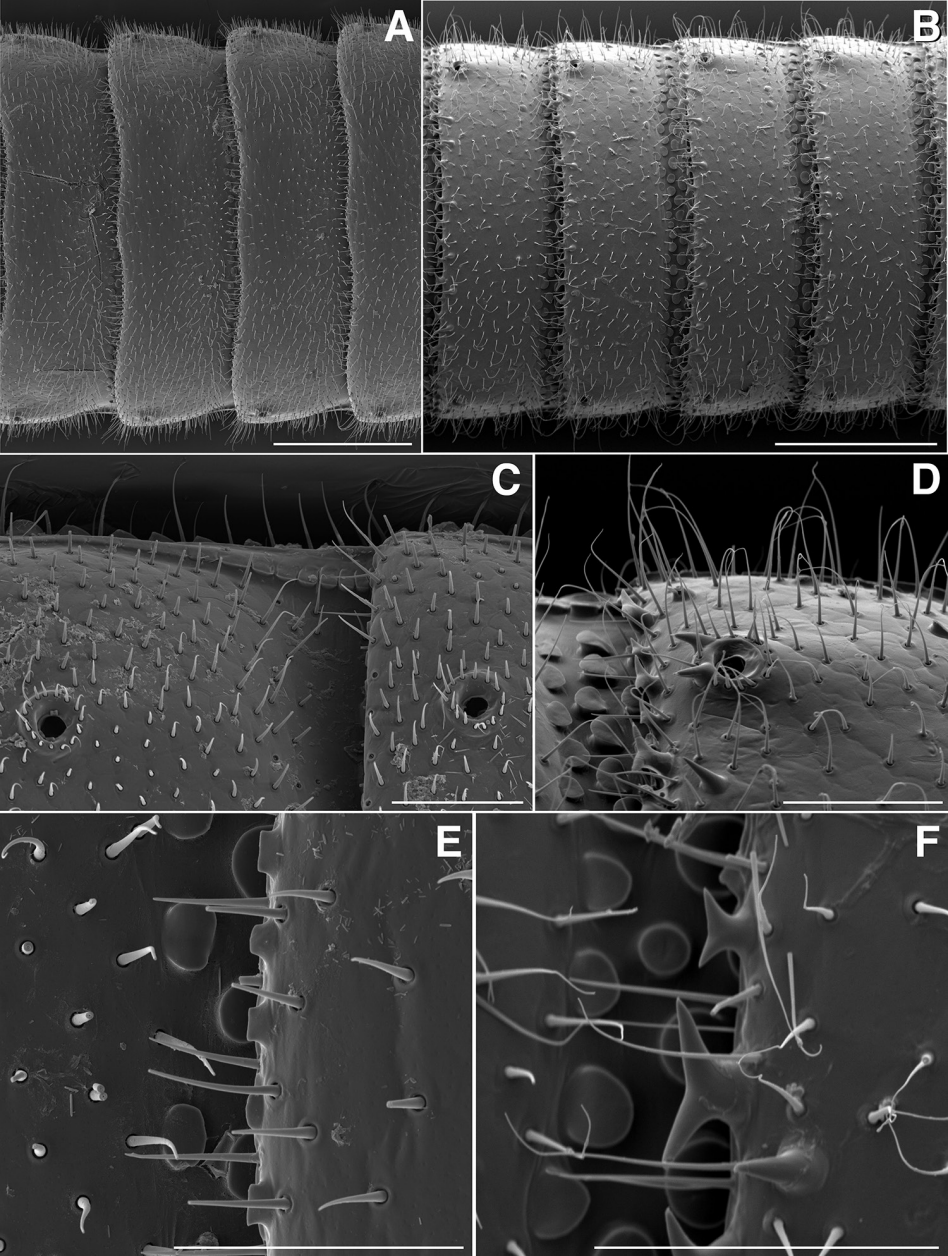
**A** Dorsal view of trunk of *Illacme tobini* sp. n. (scale bar 200 µm) **B** the same of *Illacme plenipes* (scale bar 200 µm). **C** Dorsal view of left ozopore of *Illacme tobini* sp. n. (scale bar 50 µm) **D** the same of *Illacme plenipes* (scale bar 50 µm) **E** Dorsal view of metazonite posterior margin (limbus) of *Illacme tobini* sp. n. (scale bar 40 µm) **F** the same of *Illacme plenipes* (scale bar 50 µm). (Catalog #s: *Illacme tobini* sp. n. MPE00735, *Illacme plenipes* SPC000932.)

**Figure 11.**
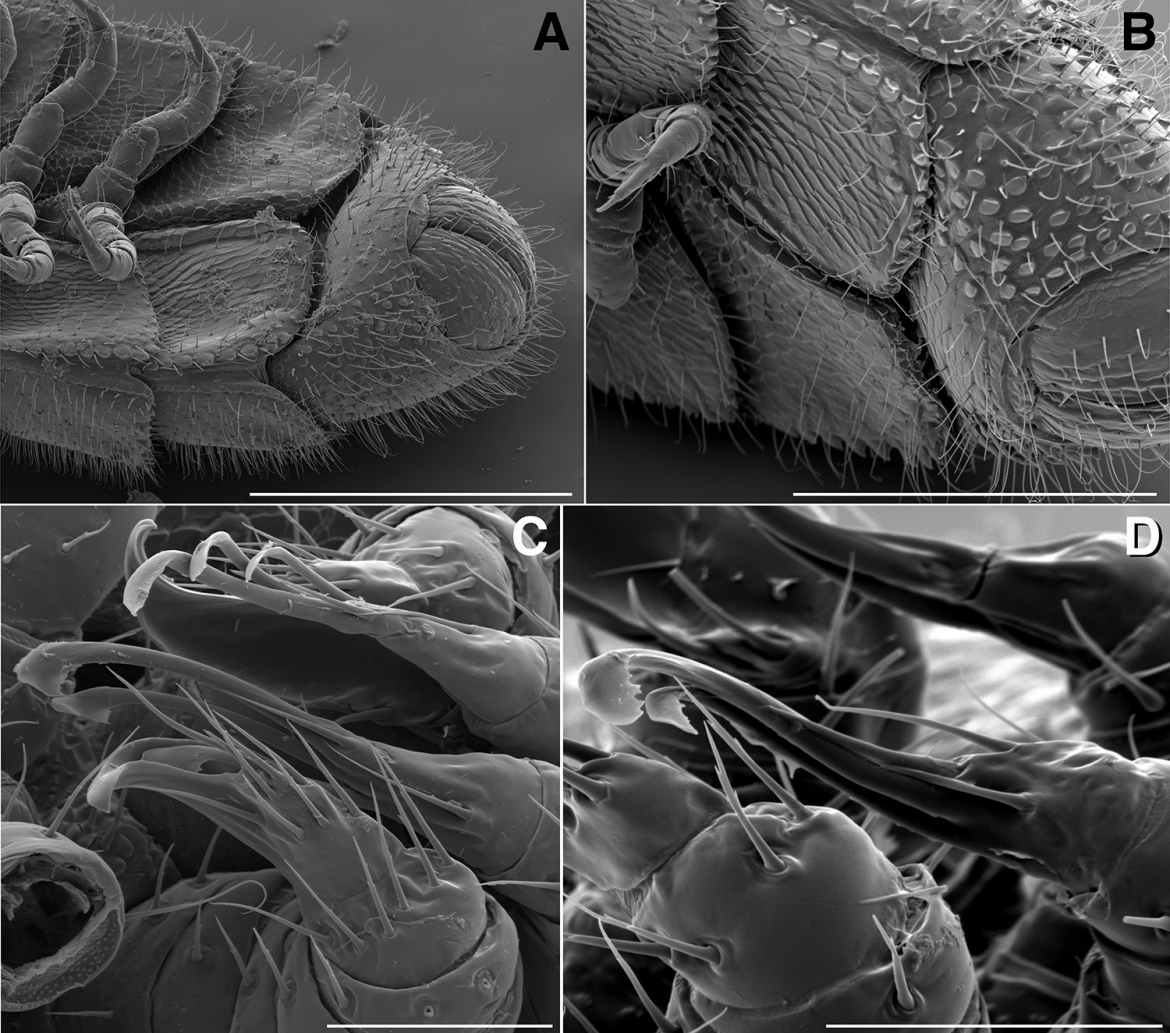
**A** Ventrolateral view of apodous ring, telson, hypoproct and paraprocts of *Illacme tobini* sp. n. (scale bar 300 µm) **B** the same of *Illacme plenipes* (scale bar 200 µm) **C** Lateral (right) view of gonopods—centered on right posterior gonopod—of *Illacme tobini* sp. n. (scale bar 50 µm) **D** the same of *Illacme plenipes* (scale bar 50 µm). (Catalog #s: *Illacme tobini* sp. n. MPE00735, *Illacme plenipes* SPC000932.)

**Figure 12.**
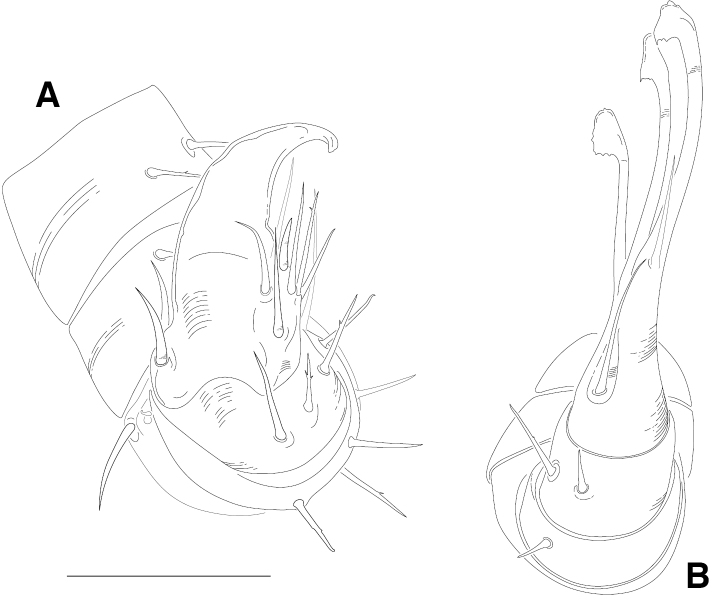
*Illacme tobini* sp. n.: **A** spade-shaped anterior gonopod (leg pair 9) **B** stylus-shaped posterior gonopod (leg pair 10). Scale bar 50 µm. (Catalog #: *Illacme tobini* sp. n. MPE00735.)

**Figure 13.**
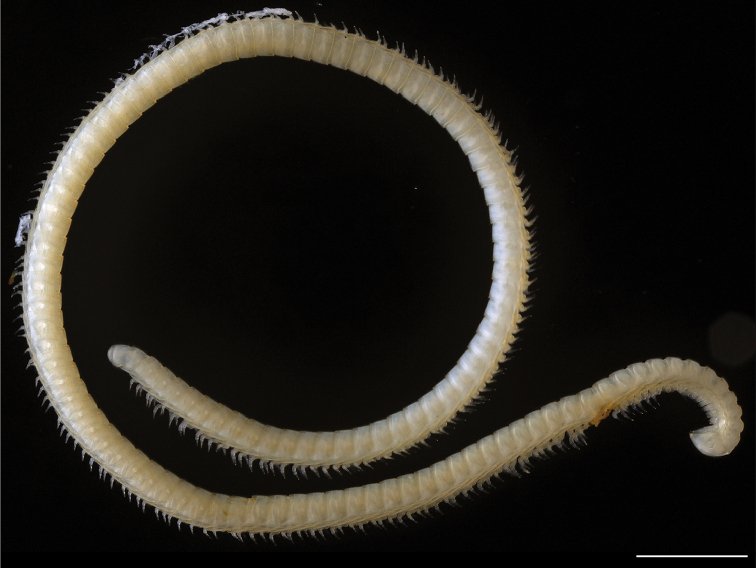
*Illacme tobini* sp. n.: ♂ holotype. Scale bar 1 mm. (Catalog #: MPE00735.)

**Figures 14.**
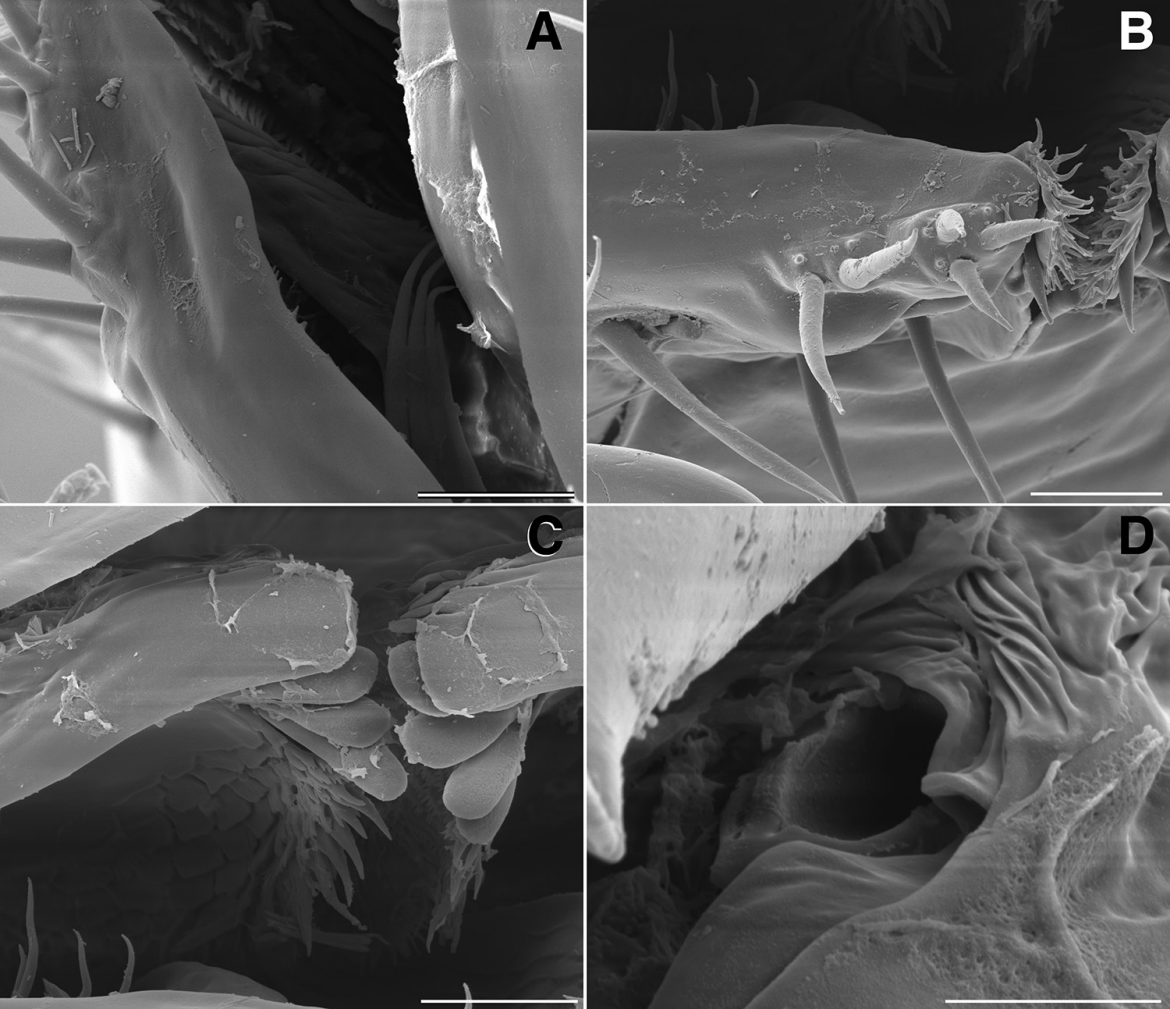
*Illacme tobini* sp. n.: **A** anterolateral (left) view of gnathochilarial stipes and dorsal surface of endochilarium (scale bar 10 µm) **B** anterior view of gnathochilarium with inner and outer palps (inner, outer palps with 3, 2 setae respectively) (scale bar 10 µm) **C** anterolateral (right) view of open mouth and keel-shaped pectinate lamella of the mandible (scale bar 10 µm) **D** anterolateral (right) view of mandible with base of external teeth a circular socket (scale bar 4 µm). (Catalog #: *Illacme tobini* sp. n. MPE00735.)

**Figure 15.**
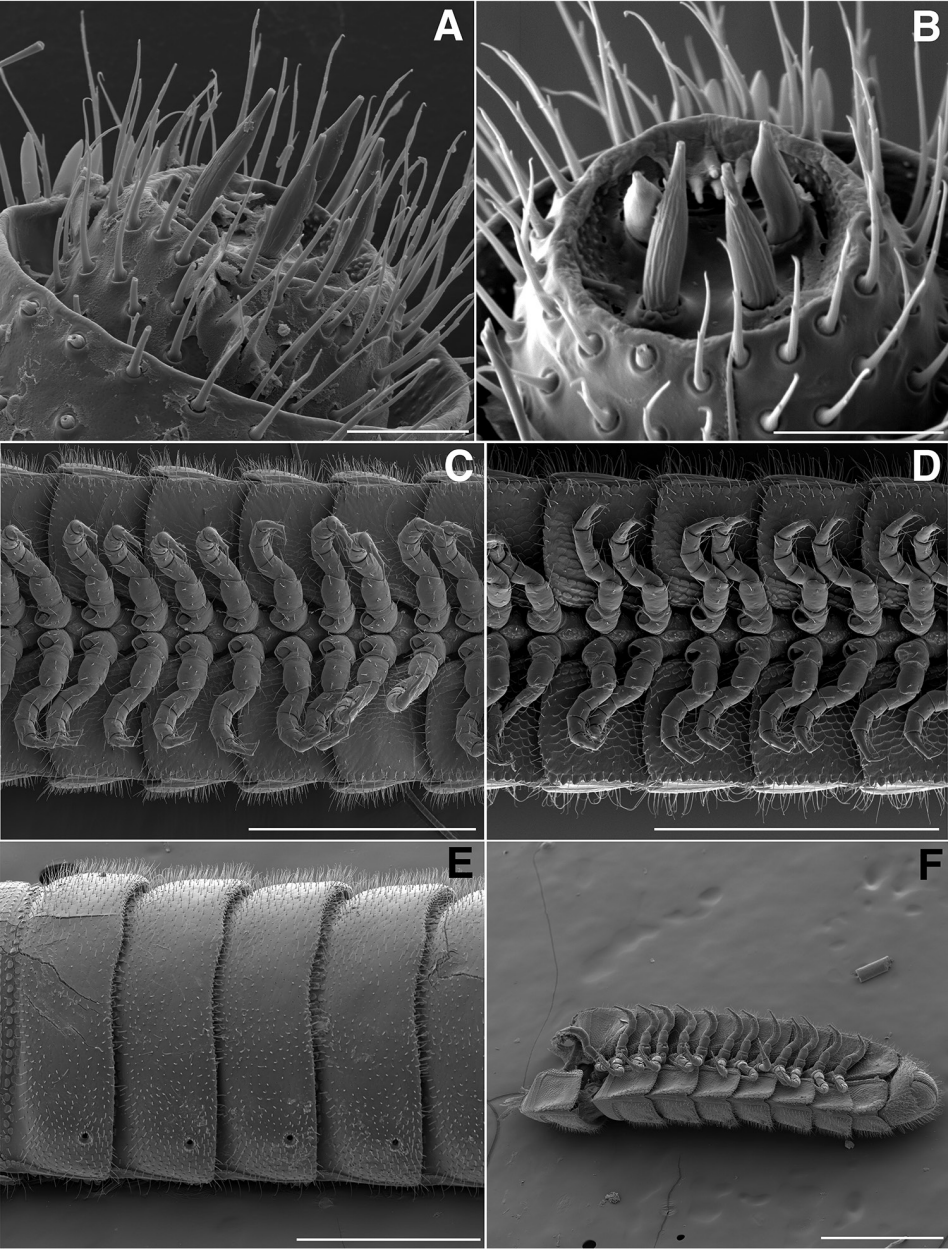
**A** Antennomere 7 of *Illacme tobini* sp. n. (scale bar 20 µm) **B** the same of *Illacme plenipes* (scale bar 20 µm). **C** Ventral view of rings of *Illacme tobini* sp. n. (scale bar 400 µm) **D** the same of *Illacme plenipes* (scale bar 400 µm). *Illacme tobini* sp. n.: **E** dorsolateral (left) view of rings 10–14 (scale bar 300 µm) **F** ventrolateral (right) view of posterior rings (scale bar 500 µm) (Catalog #s: *Illacme tobini* sp. n. MPE00735, *Illacme plenipes* SPC000932.)

**Figure 16.**
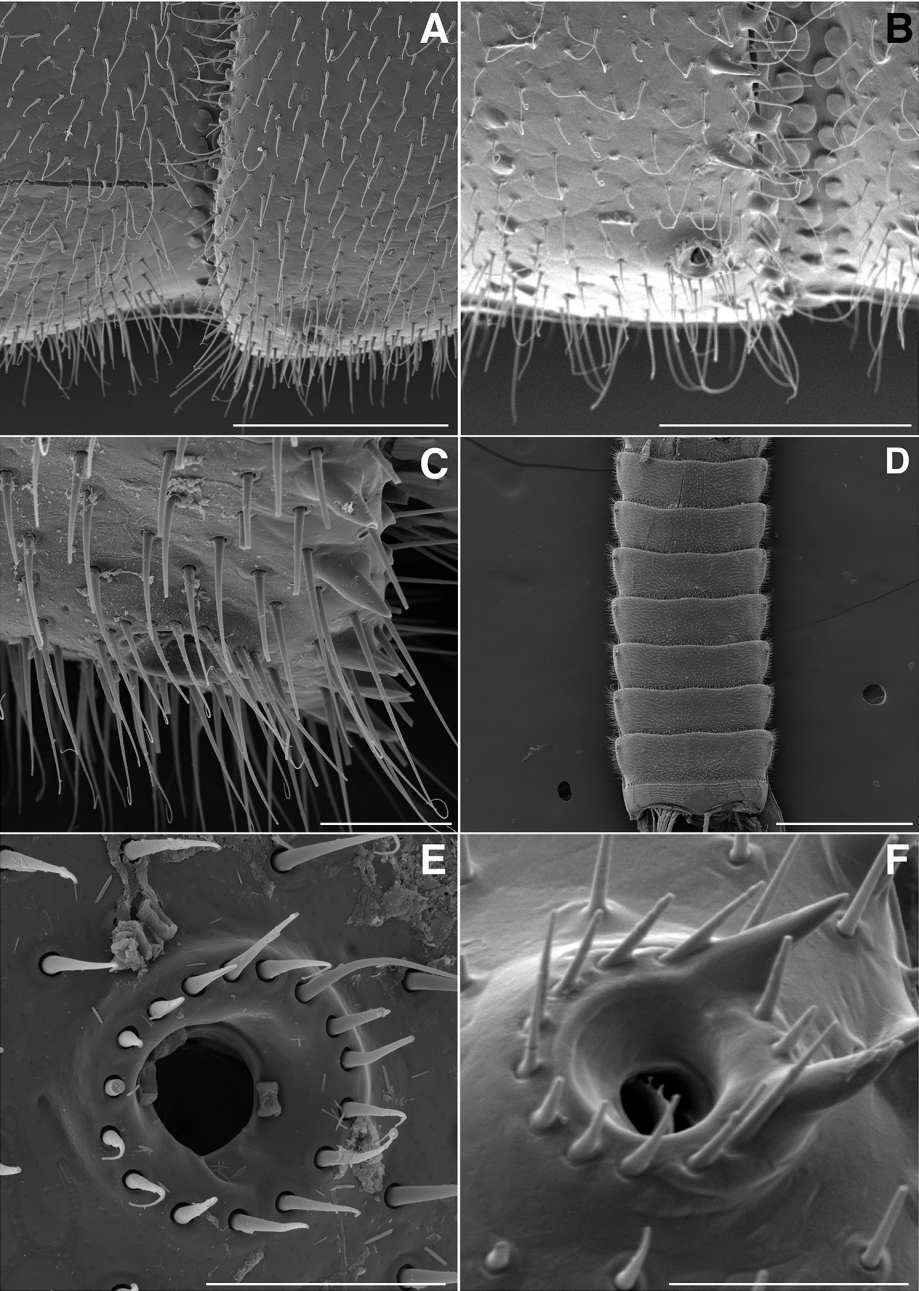
**A** dorsal view of right ozopore of *Illacme tobini* sp. n. (scale bar 100 µm) **B** the same of left ozopore of *Illacme plenipes* (scale bar 100 µm). *I tobini* sp. n.: **C** lateral (right) view of right ozopore from ring 106 (scale bar 20 µm) **D** dorsal view of trunk (scale bar 500 µm) **E** Ozopore of *Illacme tobini* sp. n. (scale bar 20 µm) **F** the same of *Illacme plenipes* (scale bar 20 µm). (Catalog #s: *Illacme tobini* sp. n. MPE00735, *Illacme plenipes* SPC000932.)

**Figure 17.**
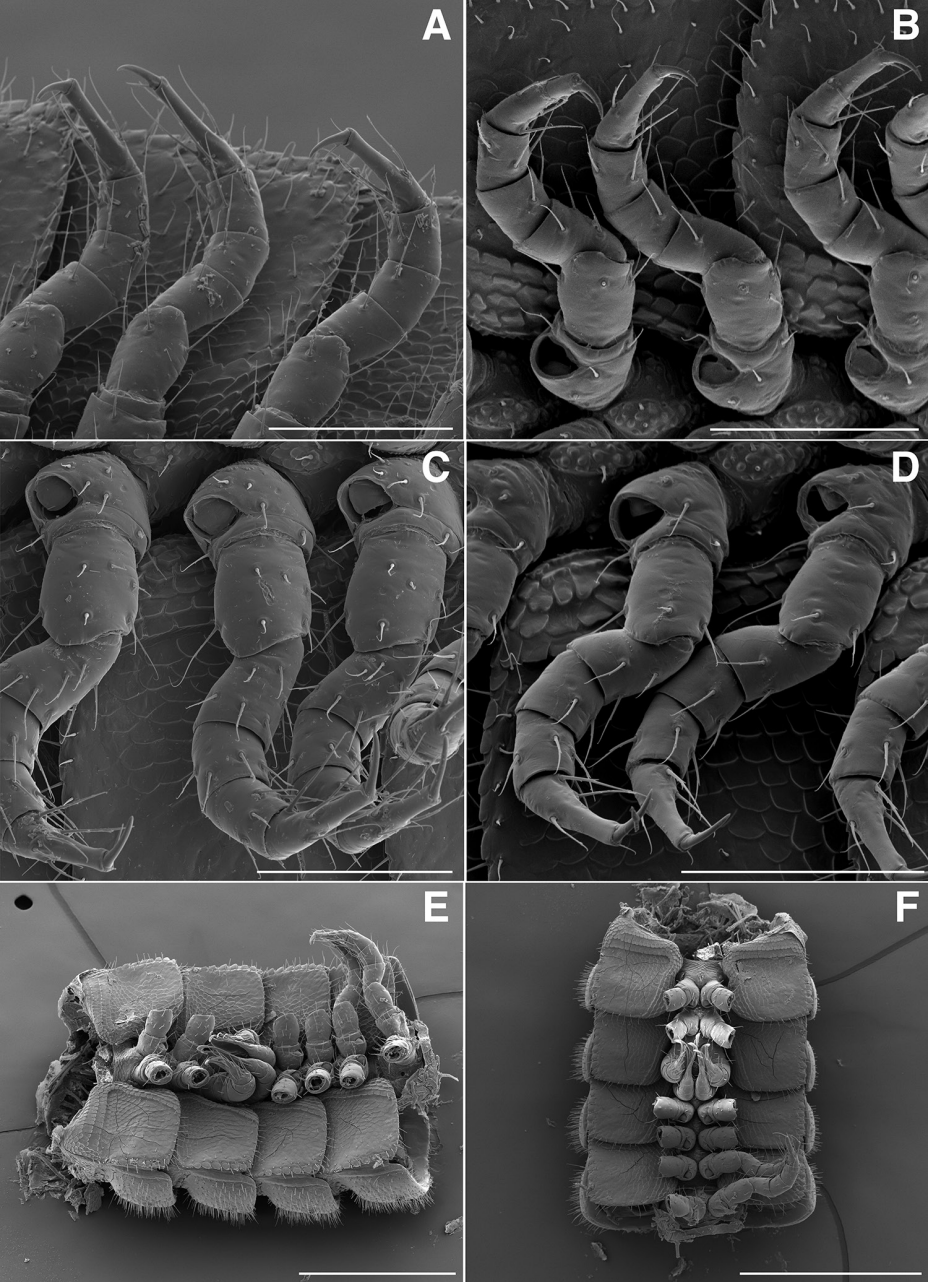
**A** Ventral view of left postgonopodal legs and claws of *Illacme tobini* sp. n. (scale bar 100 µm) **B** the same of *Illacme plenipes* (scale bar 100 µm). **C** Ventral view of left postgonopodal legs and eversible sacs of *Illacme tobini* sp. n. (scale bar 100 µm) **D** the same of *Illacme plenipes* (scale bar 100 µm). *Illacme tobini* sp. n.: **A** ventrolateral (right) view of rings 6–9 with gonopods in situ (scale bar 300 µm) **B** ventral view of the same (scale bar 400 µm). (Catalog #s: *Illacme tobini* sp. n. MPE00735, *Illacme plenipes* SPC000932.)

**Figure 18.**
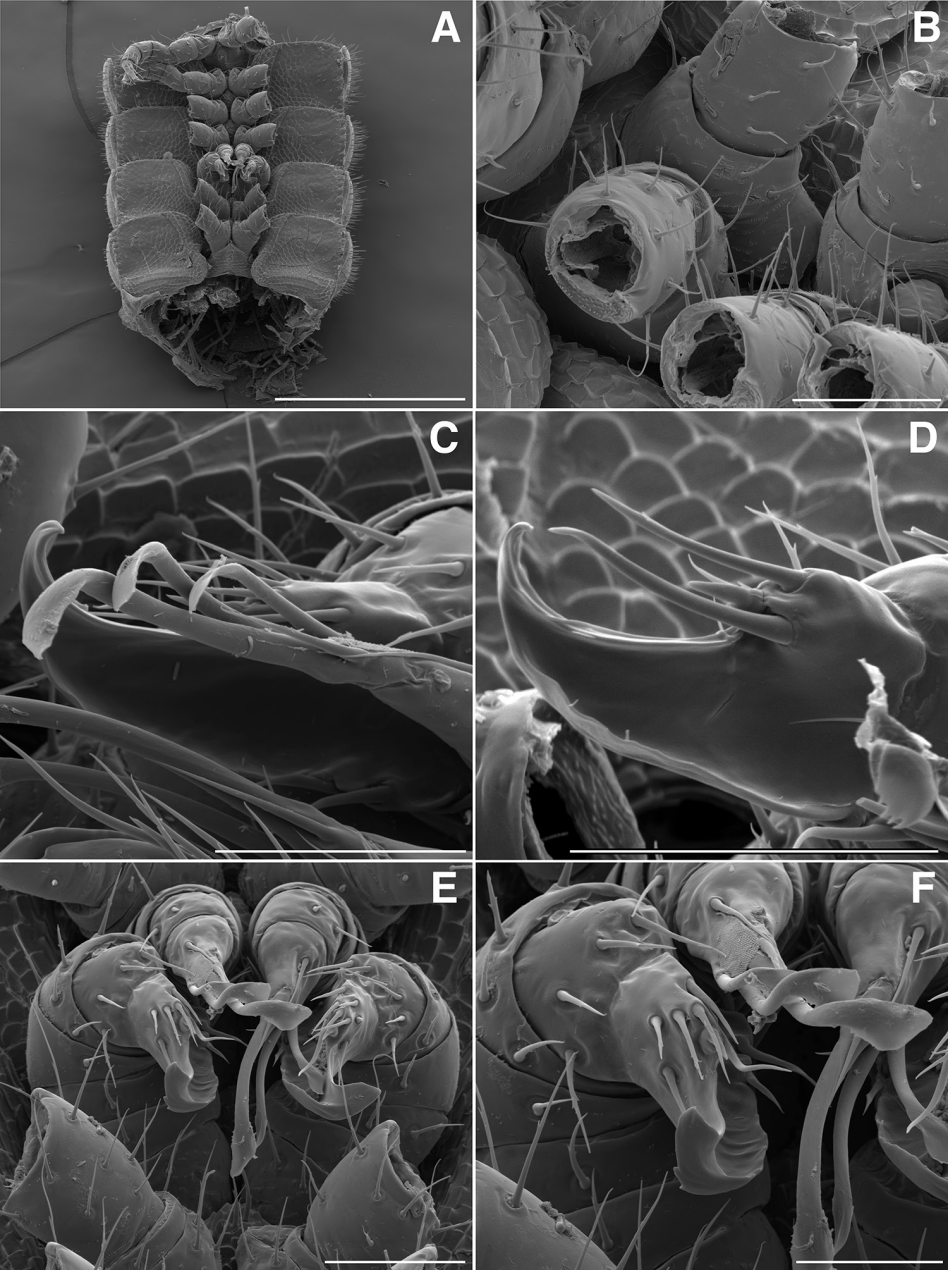
**A** Anteroventral view of rings 6–9 of *Illacme tobini* sp. n. with gonopods in situ (scale bar 400 µm) **B** ventrolateral (right) view of second leg pair with posteriorly oriented coxal gonapophyses (legs broken off at prefemur-femur joint) (scale bar 50 µm). **C** Medial view of right anterior gonopod of *Illacme tobini* sp. n. (scale bar 40 µm) **D** the same of *Illacme plenipes* (scale bar 50 µm). *Illacme tobini* sp. n.: **E** anteroventral view of gonopods *in situ* (scale bar 50 µm) **F** the same, close-up of right anterior gonopod (scale bar 30 µm). (Catalog #s: *Illacme tobini* sp. n. MPE00735, *Illacme plenipes* SPC000932.)

**Figure 19.**
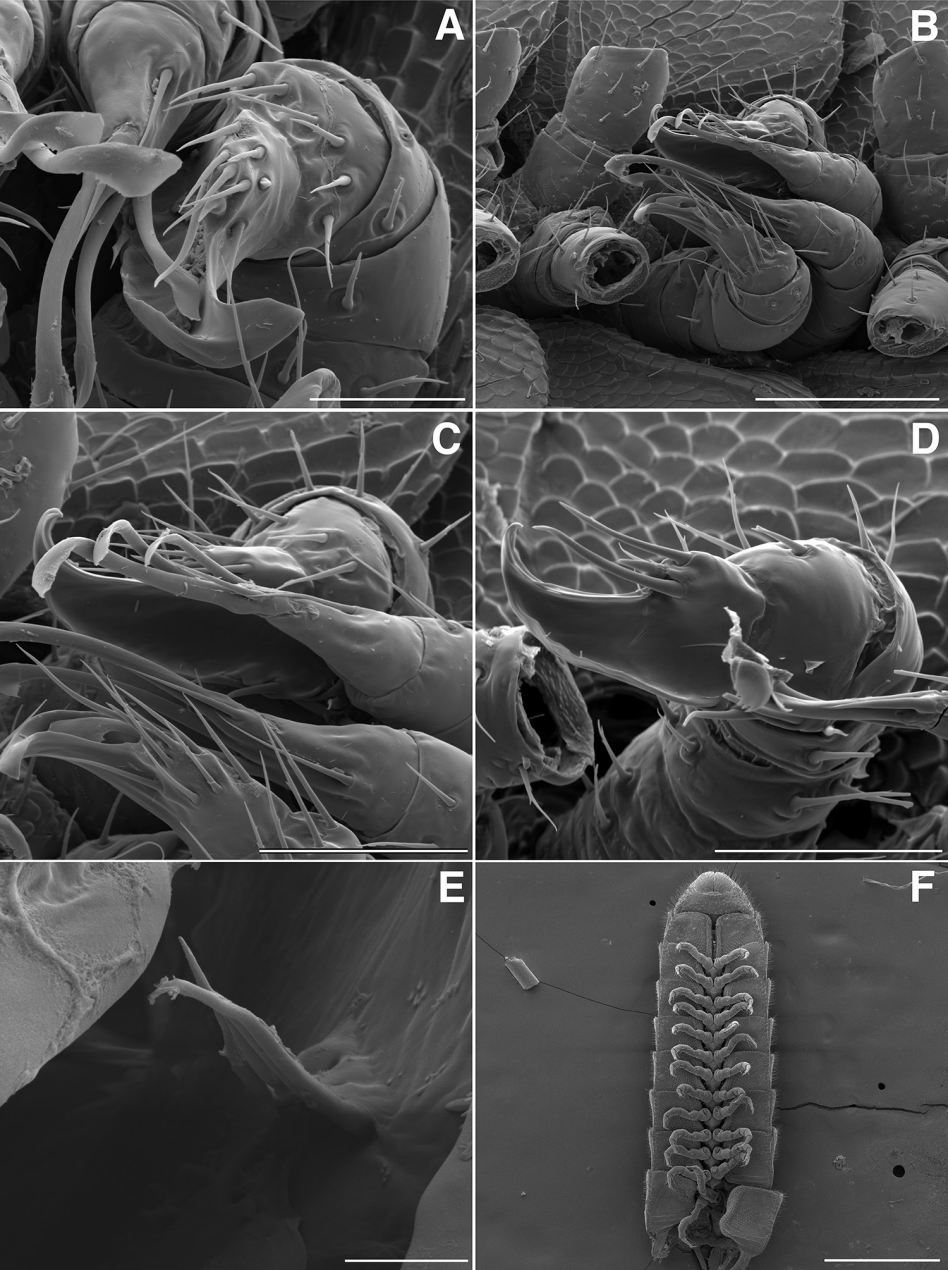
*Illacme tobini* sp. n.: **A** anteroventral view of left gonopod *in situ* (scale bar 30 µm) **B** ventrolateral (right) view of gonopods *in situ* (leg pairs 7, 8, 11 broken off at prefemur-femur joint) (scale bar 100 µm) **C** Medial view of right anterior gonopod of *Illacme tobini* sp. n. (scale bar 50 µm) **D** the same of *Illacme plenipes* (scale bar 50 µm). *Illacme tobini* sp. n.: **E** anterodorsal view of head with mouth open showing dorsal surface of left gnathochilarial stipe with unidentified brush-like structure (scale bar 5 µm) **F** ventral view of posterior rings (scale bar 500 µm). (Catalog #s: *Illacme tobini* sp. n. MPE00735, *Illacme plenipes* SPC000932.)

## Taxonomy

### Class Diplopoda de Blainville in Gervais, 1844 Subclass Chilognatha Latreille, 1802/1803 Infraclass Helminthomorpha Pocock, 1887 Subterclass Colobognatha Brandt, 1834 Order Siphonophorida Hoffman, 1980 Family Siphonorhinidae Cook, 1895

#### Genus Illacme

Taxon classificationAnimaliaSiphonophoridaSiphonorhinidae

Cook & Loomis, 1928

##### Family placement.

The genus *Illacme* is placed in the family Siphonorhinidae based on the following characters: Head pear-shaped (♂) or triangular (♀), not elongate or beak-shaped, as in the Siphonophoridae (Fig. 2A–F). Antennae elbowed between antennomeres 3, 4 (Figs 2B; 3A, B). Antennomeres 5, 6 with apical dorsal cluster of 7 or 8 basiconic sensilla (Bs_2_) in slight depression, not in defined circular pits, as in the Siphonophoridae (Figs 2B, D; 3A, B). Antennomere 1 set deep in cranium, not entirely visible dorsally as in Siphonophoridae (Figs 2A, B; 2E; 3C, D). Antennomere 2 longer than wide, conical, not doughnut-shaped and wider than long as typical in Siphonophoridae. Anterior margin of collum straight, not emarginate medially as in Siphonophoridae. Sterna with prominent midline triangular projections, oriented ventrally (Figs 3E, F; 4A, B). Posterior gonopods with distal podomere divided into 2–4 branches with one branch spike-like (Figs 4C, D, E, F; 5A–D). See also diagnoses of *Illacme* in Shelley (1996b, pg. 23), Marek et al. (2012, pg. 85), and Enghoff et al. (2015, pg. 386), and of Siphonorhinidae in Shelley and Hoffman (2004, pg. 218), Wesener (2014, pg. 417), and Enghoff et al. (2015, pg. 386).

Diagnosis. Adults of *Illacme* are distinct from other siphonorhinid genera (and commonly encountered millipedes co-occurring with *Illacme tobini* sp. n. and *Illacme plenipes*) based on the combination of the following characters: Body light cream-colored, thread-like, extremely narrow and long (max. width: ♂ 0.55, ♀ 0.64; max. length: ♂ 28.16, ♀ 40.40). Adult individuals with 84–192 segments, and with 318–750 legs. Body covered with many long delicate setae, imparting a velvety appearance (Figs 5E, F; 6A, B). Antennae elbowed between antennomeres 3, 4 (Figs 2B; 3A, B). Antennomeres 5, 6 enlarged, appearing much larger relative to other articles (Figs 2B, D, 3A, B). Head pear-shaped in males or triangular or chevron-shaped in females, eyeless (Fig. 2C–F). Genae slightly convex (♂) or straight (♀), not concave (imparting a teardrop-shaped head) as in *Nematozonium filum*, *Siphonorhinus* sp. (Wesener 2014), and the family Siphonophoridae (Shelley 1996, Shelley and Hoffman 2004). Mouthparts (gnathochilarium, mandibles) and labrum tightly appressed, tapered anteriorly to rounded apex—not beak-shaped, as in the Siphonophoridae (Figs 2A–F; 3C, D). Labrum with a deep medial slit, margins lined with teeth (Figs 6C–F; 7A–F). Denticulate shelf-like carina, projecting dorsally from labrum-epistome margin (Figs 6E, F; 7D; 8A, B). 9th and 10th leg pairs modified into gonopods, each comprising 7 podomeres (Figs 4C, D; 8C–F; 9A–F). Anterior gonopod thick, bulkier than posterior gonopod (Figs 4C, D; 8C–F). Anterior gonopodal apex (podomere 7, A7—Fig. 4C, D) spade-shaped; at rest, cupped sheath-like around posterior gonopodal stylets (podomere 7, P7—Figs 4E, F; 9A–F). Posterior gonopodal podomere 7 deeply divided, comprising a bundle of 3 (*Illacme plenipes*) or 4 (*Illacme tobini* sp. n.) stylus-shaped articles; one article spike-shaped (Fig. 4E, F); other siphonorhinid taxa with 2 stylus-shaped articles and a small spine (*Nematozonium filum*) or 2 articles without spine (*Siphonorhinus* species and *Kleruchus olivaceus* Attems, 1938). 2, 3 dorsal-most, longest articles laminate distally and recurved laterally, with denticulate posterior margins appearing saw-like (Fig. 5A–D). Ventral-most, shortest article acuminate distally, spike-like.

#### Illacme tobini

Taxon classificationAnimaliaSiphonophoridaSiphonorhinidae

Marek, Shear & Krejca, 2016 sp. n.

http://zoobank.org/65D9B6C5-A148-4CC0-8509-D32364B7034F

##### Material examined.

♂ holotype (Virginia Tech Insect Collection, VTEC catalog # MPE000735) from United States, California, Tulare County, Sequoia National Park, Lange Cave, a marble cave near intersection of Yucca and Cave Creeks, Elevation 1231 m, 9 October 2006, from within cave (Coll: J. Krejca). Exact coordinates withheld due to species rarity and habitat sensitivity.

##### Diagnosis.

Adult males of *Illacme tobini* sp. n. are distinct from *Illacme plenipes*, its sole congener, based on the combination of: Metazonites wider than prozonites with slightly enlarged paranota (Fig. 10A), not subequal in width as in *Illacme plenipes* (cf. Fig. 10B). Peritreme without the 2 large backwards projecting spines (Fig. 10C) as in *Illacme plenipes* (cf. Fig. 10D), ozopore ringed with ca. 15 setae. Ozopores nearer to margin, oriented dorsolaterally (Fig. 10A), not dorsally as in *Illacme plenipes* (cf. Fig. 10B). Metazonite posterior margin (limbus) lined with quadrate posteriorly projecting spines (Fig. 10E), not anchor-shaped as in *Illacme plenipes* (cf. Fig. 10F). Posterior margin sinuate, with anteriorly curved paramedial margins (Fig. 10A), not straight as in *Illacme plenipes* (cf. Fig. 10B). Telson densely covered with irregularly oriented and unevenly distributed stout spines on lateral surface only (Fig. 11A); telson not covered with stout spines on all surfaces and without posterior margin lined with posterodorsally oriented anchor-shaped spikes as in *Illacme plenipes* (cf. Fig. 11B). Hypoproct with two setae (Fig. 11A), not as in *Illacme plenipes* with > 2 seta present and arranged in a setal row (cf. Fig. 11B). Anterior gonopodal apex (podomere 7) spinose (Fig. 9C, E), with two-fold more spines than *Illacme plenipes* (cf. Fig. 9D, F). Anterior gonopodal podomere 3 with 2 long setae (Fig. 8E), not ringed with 6 setae, as in *Illacme plenipes* (cf. Fig. 8F). Posterior gonopodal apex (podomere 7) comprising a bundle of 4 styliform articles, with one article spike-shaped (Figs 11C, 12B), not bundle of 3 styliform articles as in *Illacme plenipes* (cf. Fig. 11D). The differential diagnosis of *Illacme tobini* sp. n. vs *Illacme plenipes* is summarized in Table 1, and a comparison of measurements between *Illacme tobini* sp. n. vs a male individual of *Illacme plenipes* (VTEC catalog # SPC000932) with an equivalent number of rings shown in Table 2.

**Table 1.** Differential diagnosis of *Illacme tobini* sp. n. versus *Illacme plenipes*.

Character	*Illacme tobini* sp. n.	*Illacme plenipes*
Rings	Metazonites wider than prozonites (Fig. 10A)	Metazonites subequal in width (Fig. 10B)
Peritreme	2 large backwards projecting spines absent (Fig. 16E)	2 large backwards projecting spines present (Fig. 16F)
Metazonite posterior margin adornment	Lined with quadrate backwards projecting spines (Fig. 10C, E)	Lined with anchor-shaped backwards projecting spines (Fig. 10D, F)
Metazonite posterior margin shape	Sinuate, with anteriorly curved paramedial margins (Fig. 10A)	Straight, without curvature (Fig. 10B)
Telson	Covered with stout spines on lateral surface only (Fig. 11A)	Covered with stout spines on all surfaces (Fig. 11B)
Hypoproct	2 setae present (Fig. 11A)	> 2 setae present, in a setal row (Fig. 11B)
Anterior gonopodomere 3	2 setae present (Fig. 8E)	6 setae present (Fig. 8F)
Anterior gonopodal apex	Spinose with two-fold more spines (Figs 9C)	Less spinose (Fig. 9D)
Posterior gonopodal apex	Bundle of 4 styliform articles (Figs 11C, 12B)	Bundle of 3 styliform articles (Fig. 11D)

**Table 2.** Comparison of measurements between *Illacme tobini* sp. n. vs a male *Illacme plenipes* individual with an equivalent number of rings (VTEC catalog # SPC000932).

	p	a	l	HW	HL	ISW	AW	CW
*Illacme tobini* sp. n.	106	2	414	0.34	0.39	0.21	0.11	0.44
*Illacme plenipes*	105	2	402	0.31	0.40	0.19	0.10	0.40
	**W1**	**L1**	**H1**	**AS1**	**A6W**	**P6W**	**BL**	***p* + *a* + *T***
*Illacme tobini* sp. n.	0.52	0.20	0.31	0.43	0.04	0.03	19.73	106 + 2 + T
*Illacme plenipes*	0.40	0.16	0.40	0.43	0.05	0.04	17.12	105 + 2 + T

##### Description of holotype

(♂) (Fig. 13). Counts and measurements: p = 106. a = 2. l = 414. (106 + 2 + T). BL = 19.73. HW = 0.34. HL = 0.39. ISW = 0.21. AW = 0.11. CW = 0.44. W1 = 0.52. L1 = 0.20. H1 = 0.31. AS1 = 0.43. A7W = 0.04. P7W = 0.03. Head pear-shaped, tapered anteriorly to round point at a 120° angle from antennal sockets; occiput gradually curved medially towards cervical area (Figs 2C, 5F, 13). Head covered with long, slender setae (Figs 2C, E, F; 3C, D). Gnathochilarium, labrum tightly appressed, tapered anteriorly to round point (Figs 2C, E, F; 3C, D, 6E, F). Mandibles not externally visible. Labrum with tooth-lined slit (Figs 6C, E, F; 7A–F). Labrum at base of slit with deeply-incised tridentate projection (Fig. 7E). Labrum posterior to slit with ca. 200 unevenly distributed pores, some with unidentified secretion extruded from the opening (Fig. 7F). Denticulate shelf-like carina, projecting dorsally from labrum-epistome margin (Figs 6E, 7D, 8A). Gnathochilarium, mandible, head capsule noticeably separate at base (Fig. 2E, F). Mandibular stipes concealed, commissure between gnathochilarium, head capsule visible distally (Fig. 2E, F). Gnathochilarium thin, plate-like, occupying three-quarters ventral length of head. Gnathochilarium tightly appressed to the ventral surface of the head, leaving a small opening anteriorly between labrum, gnathochilarial stipes. Lateral opening apparent between gnathochilarium and head capsule (Figs 2C, E, F; 3C, D). Gnathochilarium with reduced sclerites: stipes, mentum, lamellae linguales present; cardines absent (Fig. 3D). Stipes of gnathochilarium with inner, outer palps (Figs 7C, D; 14B). Lamellae linguales with palps (Fig. 14B). Mandibles not externally visible, mandibular cardo base noticeable between head capsule, gnathochilarium (Figs 2E, 3D, 5E). Mandible with ca. 5 flabellate external teeth, pectinate lamella with numerous rows of jagged ventrally projecting serrulae, nested in groove of endochilarial frontal body (Figs 7A–D; 14C). (Alternative description, primary homology with epipharynx: Epipharynx with distal flabellate side lobes, spiniferous keel with zipper construction. Mandibles, as in *Illacme plenipes* thin, stylet-like, with heavily calcified apices—not apparent externally, only visible at 400× through translucent head capsule with phase-contrast imaging on a compound microscope). Mandible (or epipharyngeal) keel nested in groove of endochilarial frontal body. Endochilarium with V-shaped frontal body (Fig. 7C, D). Endochilarium with fringed lobes (Figs 7C, D; 14B). Endochilarial fringed lobes (spatulae sensu Silvestri, 1903) protruding distally through gnathochilarial stipes and lamellae linguales (Figs 7C, 14B). Antennae sub-geniculate, elbowed between antennomeres 3, 4, comprising 7 antennomeres (Fig. 3A). Antennomeres 5, 6 enlarged. Five sensillum types: 4 apical cones (AS) oriented in a trapezoidal cluster on 7th antennomere, with longitudinally grooved outer surface and circular pore apically (Fig. 15A). Chaetiform sensilla (CS) widely spaced on antennomeres 1–7, each sensillum with 2 or 3 barbules (Fig. 3A). Trichoid sensilla (TS) oriented apically encircling antennomeres 1–7, lacking barbules (Fig. 3A). Small basiconic sensilla (Bs_2_) in clusters of 3 and 4 oriented apical dorsally (retrolaterally) on antennomeres 5 and 6; smooth, capsule-shaped, 1/2 length of chaetiform sensillum (Figs 3A, 15A). Spiniform basiconic sensilla (Bs_3_) in cluster of 4, oriented apical dorsally on 7th antennomere; tips facing apical cones (on longitudinal axis with Bs_2_ on antennomeres 5, 6); each sensillum with 3–5 barbules (Fig. 15A). Antennae extend posteriorly to middle of 3rd tergite. Relative antennomere lengths 6>2>5>3>4>1>7. Collum not covering head, with straight cephalic edge, gradually tapering laterally (Figs 2A, C; 5E). Lateral margin of collum round, with thickened scaly carina (Figs 3D, 5E). Carina repeated serially on lateral tergal and pleural margins (absent from telson). Lateral tergal and pleural carinae jagged, pronounced on midbody segments (Fig. 15C, E). Metazonites wider than prozonites, with slightly enlarged paranota (Fig. 10A). Metazonites trapezoidal, anterior margin 3× wider than long, posterior margin 3.5× wider than long. Metazonites slightly convex (Figs 6A, 15E). Metazonite dorsally covered with long, slender setae (Figs 6A; 10A; 16A, C). Tergal setae hollow, cavity diameter at base 1/4 that of setae diameter; tipped with silk-like exudate, tangled, appearing adhered to neighboring setae (Figs 6A; 16A, C). Metazonite posterior margin (limbus) lined with quadrate posteriorly projecting spines, not anchor-shaped, with row of spines anterior to limbus on posterior rings only (Figs 10E; 15E; 16A, C, D). Limbal quadrate spikes uniform in size along margin. Ozopores oriented dorsolaterally, located near lateral metazonal margin, 1/4 length of metazonite anteriorly from limbus (Fig. 10A). Ozopores absent from collum, tergites 2–4, and telson. Ozopores elevated slightly on peritremata (porosteles absent), without 2 large backwards projecting spines, encircled with ca. 15 robust setae (Figs 10C; 16A, C, E). Without lunate-arranged stout flat tubercles encircling ozopore. Posterior tergites more convex, covered with a greater density of long, slender setae (Figs 11A, 15F, 16C). Apodous segment lacking sternum, pleurites contiguous in midline. Apodous tergite densely setose, without vestiture of spikes (Fig. 11A). Telson covered with irregularly oriented and unevenly distributed stout spines on lateral surface only; without posterodorsally oriented anchor-shaped spikes (Fig. 11A). Prozonite highly sculptured, with ca. 12 rows of discoidal flat tubercles; anterior 9 rows aligned and posterior 2 rows staggered (Figs 15E, 16D). Prozonal posterior discoidal tubercles button-shaped protuberant, anterior tubercles flush with surface. Pleurites quadrate, flat, with jagged scaly lateral, posterior and medial margins (Fig. 15C). Pleurite medial margin broad, with scaly carina (Figs 3E; 8C; 15C; 17A, C). Pleurites plate-like, left and right combined comprising four-fifths of ventral segment area. Pleural medial margins broadly overlapping sternite, covering spiracles (Fig. 3E, 4A, 15C). Anterior, posterior sternites free, separate from pleurites; heart-shaped, wider anteriorly (Figs 17E, F; 18A). Sternum with prominent midline triangular ridge projecting ventrally, with spiracles and legs oriented ventrally (Figs 3E; 4A; 15C; 17E, F; 18A). Spiracles circular, orifice open; oriented dorsal to legs (Figs 3E, 4A, 17E). Tergites, pleurites and sternites separated by arthrodial membrane (Figs 11A; 15C, F; 17A). Arthrodial membrane between tergites and pleurites wider posteriorly, pleated (likely permitting telescoping body rings). Telson covered with long slender posteriorly curved setae (Fig. 11A). Paraprocts semihemispherical, anterior margins slightly scaly (Fig. 11A). Hypoproct small, one-eighth area of paraproct, with two posterior projecting setae. Legs with six subequally shaped podomeres, with coxa slightly shorter and tarsus slightly longer. Legs with sparse setae, appearance similar to trichoid sensilla, with 2 or 3 barbules. Coxae nearly contiguous medially, separated by thin sternal ridge. Large posteroventral D-shaped opening for eversible sac (Figs 3E, 8C, 15C, 17C). Eversible sacs membranous, bulging slightly within aperture (Figs 3E, 17C). Tarsus with pincer-like claw; dorsal claw arcuate, ventral accessory seta thick, stout (Figs 2A, C, F; 6E, 17A). 2nd leg pair with posteriorly oriented coxal gonapophyses; rounded, protuberant, one-half length of prefemur (Fig. 18B). 9th, 10th leg pairs modified into gonopods, each comprising 7 podomeres (Figs 4C, E; 8C, E; 9A, C, E; 12A, B). Anterior gonopod robust, thicker than posterior gonopod (Figs 4C, 8E, 12A). Anterior gonopodal apex (podomere 7) shovel-shaped; in repose cupped around flagelliform posterior gonopodal apex (podomere 7, Figs 4C, E; 8E; 9A, C; 12A). Posterior gonopodal podomere 7 deeply divided, comprising a bundle of 4 stylus-shaped articles (Figs 4C, E; 5A, C; 8E; 9A, E; 12B; 18C, E, F; 19B, C). 3 dorsal-most, longest articles laminate distally, recurved laterally, denticulate posterior margins (Figs 5C, 12B). Ventral-most, 4th article acuminate distally, spike-like (Figs 4E, 12B). Thin ridge-shaped sterna present between left and right gonopods, thicker between anterior gonopods. Supplementary micrographs of *Illacme tobini* sp. n. are archived in the Dryad Data Repository at https://doi.org/10.5061/dryad.tk0b8.

Female unknown.

##### Etymology.

This new species is named for Ben Tobin, Cave Specialist and Hydrologist at Grand Canyon National Park. Ben organized and carried out numerous cave surveys in the U.S., including the field visit that uncovered *Illacme tobini* sp. n., and has facilitated the discovery of many new species of invertebrates and other cave fauna in Sequoia National Park. The specific name is a genitive noun derived from his surname.

##### Variation.

Unknown. *Illacme tobini* sp. n. is known from a single male specimen (Fig. 13).

##### Habitat and distribution.

**Figure 20.**
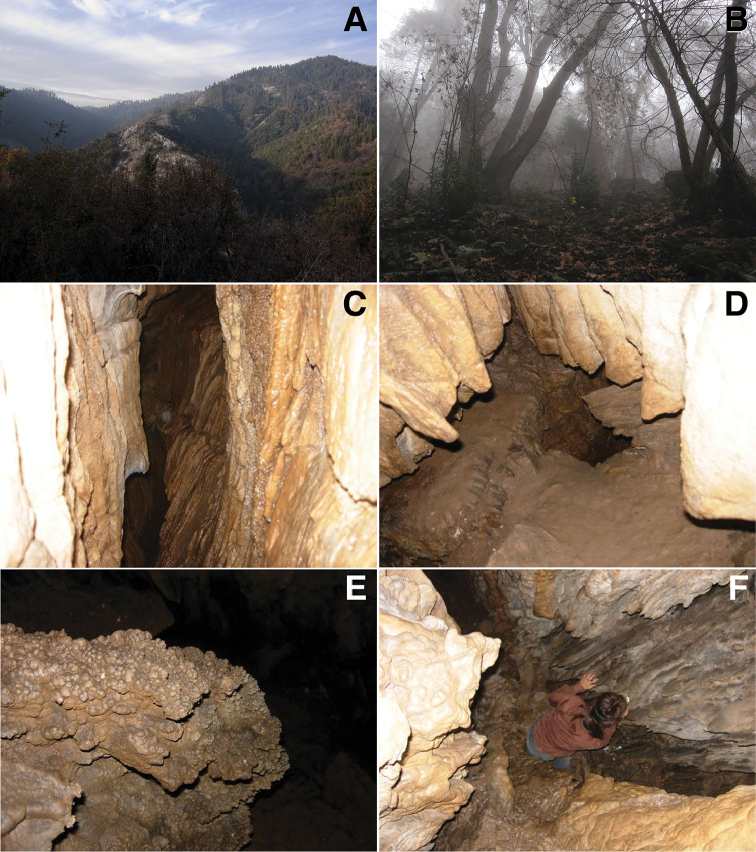
Habitat of *Illacme tobini* sp. n.: **A** Yucca Ridge, Sequoia National Park, California, with marble rocks exposed along ridge (center) **B** Woodland habitat on north facing slope of Yucca Canyon **C–F** Interior of marble cave.

*Illacme tobini* sp. n. is only known from a single in-cave collection, within the upper foothills of the Giant Forest in Sequoia National Park (Fig. 1B). Lange Cave is situated at the base of Yucca Mountain at the boundary of the Sierra Nevada Forest and California Interior Chaparral and Woodlands ecoregions (Fig. 20). A region characterized by a Mediterranean climate with temperature and humidity extremes encompassing cold wet winters (< 0 °C and 700 mm precipitation) and hot dry summers (> 40 °C and < 2 mm precipitation) (Tobin et al. 2013). The cave is composed of Jurassic-Triassic marble of a white, coarsely crystalline, and schistose to gneissose composition (Sisson and Moore 1994). The marble cave system is encompassed by biotite-feldspar-quartz schist rocks. The cave is ca. 90% surveyed, and has a total volume of 354.2 m^3^, average diameter of 2.1 m, wall area of 733.3 m^2^, and floor area of 124.6 m^2^. Inside the cave, temperatures range between ca. 6 °C in the winter months (October–May) to ca. 9 °C in the summer months (June–September). The woodland habitat around the cave was primarily composed of California live-oak (*Quercus agrifolia*), California bay (*Umbellularia californica*), Giant sequoia (*Sequoiadendron giganteum*), and Mountain maple (*Acer glabrum*). Understory flora included Scouringrush horsetail (*Equisetum hymale*), California wood fern (*Dryopteris arguta*), and Thimbleberry (*Rubus parviflorus*). Other organisms encountered in the habitat included millipedes—*Parcipromus cooki*, *Californiulus yosemitensis*, *Taiyutyla loftinae*, *Amplaria muiri*; arachnids—*Yorima* sp., *Ceratinops inflatus*, *Nesticus* spp., *Pimoa* spp., *Mundochthonius* sp., *Ortholasma colossus*, *Calicina* sp.; hexapods—*Tomocerus* sp., *Amoebaleria caesia*, *Heleomyza* sp., *Hippodamia* convergens; and the salamander *Ensatina eschscholtzii platensis*.

##### Discussion.

*Illacme* species have extremely limited known geographic ranges. This feature suggests a formerly widespread, perhaps ancient, distribution, and/or membership in a larger hidden diversification in California encompassing many undiscovered taxa. *Illacme* individuals occur in the mesovoid shallow substratum (MSS), a cryptic ecosystem, which are miniscule subterranean microhabitats encompassing fissures and cracks below the soil surface (Ortuño et al. 2013). These subterranean areas are the microcaverns (< 1 mm) and mesocaverns (1 mm–20 cm) described by Howarth (1983). The fauna of the MSS likely represents a considerable fraction of unknown biodiversity, yet the habitat is unexplored and its diversity poorly known. Species discovery from these microhabitats has only recently begun, and recent advances in collecting techniques are uncovering a considerable amount of new taxa. These microhabitats are fundamentally miniature caves and many MSS taxa also include cave-restricted species (Espinasa et al. 2014). As a result, MSS organisms possess some troglomorphic features—e.g., lack of eyes, no pigment—but lack the open-space adaptations of cave animals, including long limbs and elongate sensory structures (e.g., antennae and setae). Frequently MSS taxa possess shorter legs than cave or epigean forms and a covering of thin, delicate setae on the exoskeleton (Espinasa et al. 2014). Albeit anecdotally, Manton (1961, pg. 395) associated the hirsute covering of *Siphonophora* individuals as an adaptation for maneuverability, including spiraling within narrow crevices, and crawling upside-down on the ceilings of caverns; however, it is unclear precisely how this occurs biomechanically. *Illacme plenipes* individuals are found exclusively beneath large deep-set stones—a common place of discovery for MSS arthropods (Marek et al. 2012). In contrast, *Illacme tobini* sp. n. was documented solely from a marble cave. Considering that *Illacme plenipes* individuals were found in the MSS, and that they possess MSS adaptations (including a vestiture of setae and absence of long sensory structures and limbs), the possibility that *Illacme tobini* sp. n.—with similar adaptations—is cave-restricted is uncertain. Additional material of *Illacme tobini* sp. n. would provide evidence to address this claim. Notwithstanding the paucity of material, the significance of the discovery highlights the importance of the Sequoia caves and MSS as a habitat of distinctive biodiversity.

The species *Illacme tobini* sp. n. and *Illacme plenipes* are the sole members of the genus and family in the Western Hemisphere. Shared morphological characters indicate the family is monophyletic, yet these features have not been considered within the context of a rigorous phylogenetic systematic framework. The features, including lack of a beak and absence of antennal pits, are broadly distributed across helminthomorph millipedes and are potentially shared ancestral traits and thereby do not indicate monophyly. The species *Illacme tobini* sp. n. is closely allied to *Illacme plenipes* based on unique shape of the head, consistency in appearance of mouthparts, similarly shaped gonopods, and possession of many legs. However, *Illacme tobini* sp. n. differs from *Illacme plenipes* in noteworthy characters such as the shape of metazonites, ornamentation of the ozopore, and chaetotaxy and number of articles of the posterior gonopods. The divergence in these traits, considering the usual divergence in morphology between siphonorhinid taxa, suggests generic differences. Our PCR possibly failed because specimen preservation in dilute ethanol degraded the DNA (Vink et al. 2005). Pending discovery of additional individuals suitable for DNA sequencing, and phylogenetic analysis within the context of relatives in the Siphonorhinidae, a new generic designation may be justified.

Our knowledge of the cephalic morphology of the Colobognatha is limited due to their small size and derived anatomy, thereby making the generation of homology hypotheses difficult. In the current study, we revise the morphological assessment of the labrum, gnathochilarium, and mandibles. The labrum of *Illacme tobini* sp. n. is apically deeply divided into a slit. The dorsal margins of the slit are lined with sharp upwards-projecting spines. Moving posteriorly into the head, the labrum is further divided into a tridentate projection with additional upwards projecting spines. The remainder of the labrum posterior to the epistome is covered in a field of ca. 200 pores, half of which possess a secretion seemingly extruded from the pore openings (Fig. 7F). These labral features are not observed in other diplopods, and their homology and function is unclear. The pores appear deep and may open to the buccal cavity. The gnathochilarium of *Illacme plenipes* was described as “indistinguishably fused” (Marek et al. 2012, pg. 89). However, we now think the gnathochilaria of both species are composed of a mentum and pair of stipes. *Illacme tobini* sp. n. has paired lamellae linguales each with a palp (Fig. 14B), but whether this feature is present in *Illacme plenipes* is unclear. The mandibles of *Illacme tobini* sp. n. lack sharp teeth distally (as in other Chilognatha) and possess finger-like rounded teeth. The mandibular pectinate lamella are composed of numerous rows of small jagged teeth that project ventrally and nest in a groove in the frontal body of the endochilarium. It is evident that these structures are the mandibles and not the epipharynx based on the articulated nature of the articles and separation between the mandibular gnathal lobe and base. In the description of *Kleruchus olivaceus*, Attems (1938, pg. 296, fig. 195) similarly illustrates the right mandible of the male holotype. In the drawing, the finger-like mandibular teeth and pectinate lamella are strikingly similar in appearance to the equivalent structures in *Illacme tobini* sp. n.

Based on examination of the mouthparts of *Illacme* and other siphonophoridan species, individuals consume liquid or gelatinous foods. In Siphonophorida (and most Colobognatha), the mouthparts are drawn into a cone with a small aperture distally. The mandibles are reduced and are not divided between the cardo, stipes and galea. A suctorial feeding mode has been suggested previously, and Manton (1961, pg. 386) indicated that species of *Siphonophora* possess a “suctorial fore-gut”, proboscis, skeleto-muscular features, and head movement behaviors that strongly implicate suction feeding. Manton did not elaborate upon precise modifications of the foregut for suction, but filtration devices and muscular thickening are potential features to explore in the future. The presence of a coiled hindgut and elongation of the trunk (and thereby also the gut) for processing a nutrient poor diet are consistent with plant sap feeding (Marek et al. 2012). Dissecting individual hindguts of *Illacme plenipes* suggested a liquid diet since gut contents were gelatinous and homogenous, and lacking particles completely. The mouthpart morphology of *Illacme tobini* sp. n. is peculiar and hypothetically represents a morphology adapted for consuming fungus as they are similar in gross anatomy with some sporophagous beetles (Betz et al. 2003, Lipkow and Betz 2005, Yavorskaya et al. 2014). Specifically, *Illacme tobini* sp. n. possesses (1) mandibles with inner brush-like “bristle-trough” structures (Fig. 14C—mouthpart feature *ii* of Lipkow and Betz 2005) that hypothetically sweep in loose food material; (2) mandibles with outer lobes for manipulating dispersed food material and transporting it posteriorly (Figs 7A–D, 14C—feature *iii* of Lipkow and Betz 2005); and (3) a flat crushing or grinding surface of the endochilarium (Fig. 7C, D—feature *iv* of Lipkow and Betz 2005).

While the mouthparts of the Siphonophorida are more derived in morphology and function relative to other helminthomorph millipedes, the gonopods are primitive due to their leg-like structure. In contrast with gonopods of eugnathan millipedes, many of which possess two leg podomeres (coxa and telopodite), colobognaths typically have a greater number of podomeres. Although Marek et al. (2012) counted six gonopodal podomeres in *Illacme plenipes*, we have reexamined *Illacme plenipes* males and found that they, as with *Illacme tobini* sp. n. males, have seven gonopodal podomeres, representing the primitive complement, including a seventh tarsungulum that is the terminal article. As in other colobognaths, the tarsungulum of the posterior gonopod is stylus-like, and the anterior tarsungulum spade-shaped with a deep groove. The groove of the anterior gonopod cups the stylus, which is often observed resting within the recess, and may act as a conductor allowing the posterior gonopod to slide into the cyphopods of the female during copulation. This process may be functionally analogous to the spider embolus (=posterior gonopod in millipedes) and conductor (=anterior gonopod in millipedes).

Several groups of dispersal-limited Californian animals show a distribution in which a Sierra Nevada clade is most closely related to a clade in the Coast Ranges. Examples of this spatial pattern occur in bioluminescent millipedes (genus *Motyxia*), harvestmen (genus *Calicina*), mygalomorph spiders (*Aliatypus californicus*, *Aliatypus erebus*), and several species of salamanders (*Batrachoseps attenuatus*, *Ensatina eschscholtzii*, *Aneides lugubris*—Jockusch and Wake 2002, Martínez-Solano et al. 2007, Kuchta et al. 2009, Lapointe and Rissler 2005). Most studies that demonstrate this biogeographical pattern among taxa infer directionality of diversification from the Coast Ranges to the Sierra Nevada (reviewed in Emata and Hedin 2016). The phylogenetic studies of salamanders indicate a west-to-east pattern, relatively recent in the mid-late Pleistocene. In contrast, the studies of mygalomorphs and diplopods recover an east-to-west directionality of diversification (Satler et al. 2011, Marek and Moore 2015). Several of these taxa (e.g., *Calicina* and *Batrachoseps*) have occurred in California since the Eocene, with a “trans-valley” split occurring in the harvestman *Calicina* during mid-late Miocene (Emata and Hedin 2016). Inferred dates for the east-west splits in other taxa are unknown.

##### Conservation.

*Illacme tobini* sp. n. is a short-range endemic restricted to the base of Yucca Mountain between the North and Marble forks of the Kaweah River in Sequoia National Park, California. The species is only known to occur in one small cave, though its range is likely to include the MSS. Management of this species should include careful consideration of activities that may impact the surface or subsurface. Actions that include vegetation changes, ground disturbance, or alteration of drainage patterns should be restricted in scope to preserve the soil and moisture of this river basin. The abundance and composition of MSS invertebrates in most global habitats remains uncertain, and further exploration and survey of these systems, thereby building knowledge of this fauna, will help to understand more fully biodiversity that is responsible for supporting healthy forests and ecosystem services.

## Species catalog of the Siphonorhinidae

**Family Siphonorhinidae Cook, 1895**

4 genera and 12 species: Wallacea, Sundaland, Himalayas, Indo-Burma, Madagascar, Maputaland-Pondoland-Albany, and North America.

Siphonorhinidae Cook 1895: 2. Jeekel 1971: 45. Hoffman 1980: 116. Shelley 1996b: 1808. Hoffman 1999: 195 (189 pdf). Jeekel 2001: 46. Enghoff et al. 2015: 386.

Indiozoniinae Verhoeff 1941: 220. Hoffman 1980: 116 (synonymized).

Nematozoniidae Verhoeff 1939: 218. Verhoeff 1940b: 506. Attems 1951: 197. Schubart 1966: 199. Jeekel 1971: 41. Hoffman 1980: 116 (synonymized).

Teratognathidae Attems 1951: 210. Hoffman 1980: 116 (synonymized).

**Genus *Illacme* Cook & Loomis, 1928**

2 species: California

*Illacme* Cook and Loomis 1928: 12. Type species: *Illacme plenipes* Cook & Loomis, 1928, by original designation. Chamberlin and Hoffman 1958: 189. Buckett 1964: 29. Jeekel 1971: 39. Hoffman 1980: 116. Shelley 1996b: 23. Shelley 1996a: 1808. Hoffman 1999: 195 (189 pdf). Jeekel 2001: 46. Marek and Bond 2006: 707. Shelley 2010: 45. Marek et al. 2012: 85. Wesener 2014: 415. Enghoff et al. 2015: 386.

***Illacme plenipes* Cook & Loomis, 1928**

*Illacme plenipes* Cook and Loomis 1928: 12. Chamberlin and Hoffman 1958: 189. Buckett 1964: 29. Enghoff et al. 1990: 131. Hopkin and Read 1992: 1. Shelley 1996b: 23, figs 1–3. Shelley 1996a: 1808. Hoffman 1999: 195 (190 pdf). Jeekel 2001: 46. Shelley and Hoffman 2004: 221. Marek and Bond 2006: 707. Read and Enghoff 2009: 554. Shelley 2010: 45. Shelley and Golovatch 2011: 26. Marek et al. 2012: 87. Wesener 2014: 415.

***Illacme tobini* Marek, Krejca & Shear, 2016**

*Illacme tobini* Marek, Krejca & Shear, 2016: herein. MALE HT (VTEC). United States: California, Tulare County, Sequoia National Park.

**Genus *Kleruchus* Attems, 1938**

1 species: Vietnam

*Kleruchus* Attems 1938: 295. Type species: *Kleruchus olivaceus* Attems, 1938, by original designation. Carl 1944: 260. Attems 1951: 211. Jeekel 1971: 40. Hoffman 1980: 116. Jeekel 2001: 46. Enghoff et al. 2015: 386.

***Kleruchus olivaceus* Attems, 1938**

*Kleruchus olivaceus* Attems 1938: 296, figs 193–203. MALE HT (NMW). Vietnam: Đà Nẵng Province, Bà Nà [15.983333 N, 107.983333 W]. Lit: *Bana (C. Annam), 1.500 m., 22 IX., 31*. Marek et al. 2012: 86.

**Note.** Attems (1938) provided illustrations of the enlarged forelegs of *Kleruchus olivaceus* (fig. 193), head and antenna (fig. 194), mandible (fig. 195), ventral surface of head and gnathochilarium (fig. 196), pleurite (fig. 198), ventral surface of the terminal 3 segments plus telson (fig. 199), and gonopods (figs 200–203).

**Genus *Siphonorhinus* Pocock, 1894**

8 species: India, Indonesia, Madagascar, Vietnam

*Siphonorhinus* Pocock 1894: 335. Type species: *Siphonorhinus pallipes* Pocock, 1894, by original designation. Jeekel 1971: 45. Jeekel 2001: 46. Enghoff et al. 2015: 386.

***Siphonorhinus angustus* Pocock, 1894**

*Siphonorhinus angustus* Pocock 1894: 336. MALE HT (BMNH). Indonesia: West Java, Bogor [-6.6 S, 106.8 W]. Lit: *Java: Buitenzorg. A single ♂ specimen*. Attems 1914: 359.

**Note.** With regards to *Siphonorhinus angustus* and *Siphonorhinus pallipes* (collected from the same area), Pocock (1894, pg. 336) wrote: “These two species are really so much alike that I am perfectly prepared for fresh specimens to show that the differences pointed out are merely due to individual variation. But at present there is no evidence that such is the case and the analogy of other species of the group lends no support to the view.”

***Siphonorhinus cingulatus* (Attems, 1936)**

*Siphonophora cingulata* Attems 1936: 315, fig. 94. FEMALE HT (NMW). Vietnam: Lâm Đồng Province, Đà Lạt [11.941667 N, 108.438333 W]. Lit: **South Annam, Dalat, 5,000 feet, Langbian Province (C. Boden Kloss; iii-v.18; 1 ex)*.* Attems 1938: 320. Carl 1941: 573. Carl 1944: 260. Turk 1947: 74.

*Pterozonium cingulatum*–Attems 1951: 231. Golovatch and Martens 1996: 167.

*Zinaceps cingulatus*–Chamberlin and Wang 1953: 13.

*Siphonorhinus cingulatus*–Jeekel 2001: 47. Golovatch and Wesener 2016: 16.

**Note.** Attems (1936) provided a second locality, lit: **India, Eastern Himalayas, Pashok, 1,500 and 2,600 feet, Darjeeling District (Dr. F. H. Gravely; 26.v.-14.vi.16; Dr. S. L. Hora; 16.xii.26; 2 exs.)** [27.075794 N, 88.408726 E].

***Siphonorhinus coniceps* (Attems, 1936)**

*Siphonophora coniceps* Attems 1936: 314, fig. 93. FEMALE HT (NMW). India: West Bengal, Darjeeling, Pashok [27.075794 N, 88.408726 W]. Lit: **India, Eastern Himalayas, Pashok, 5,000 feet, Darjeeling District (Dr. F. H. Gravely; 26.v.-14.vi.16; 1 ex.).** Turk 1947: 74.

*Indozonium coniceps*–Verhoeff 1941: 215.

*Siphonorhinus coniceps*–Carl 1941: 573. Carl 1944: 260. Jeekel 2001: 47. Golovatch and Wesener 2016: 16.

*Pterozonium coniceps*–Attems 1951: 231. Attems 1953: 198, figs 117–119.

*Zinaceps coniceps*–Chamberlin and Wang 1953: 13.

**Note.** Attems (1936) described *Siphonorhinus coniceps* and *Siphonophora cingulata* exclusively from female material. Carl (1941, 1944) and Jeekel (2001) did not provide a justification for rehousing the species within *Siphonorhinus*. The decision was likely made from Attems’s (1936, pg. 315, fig. 93a) drawing of the head of *Siphonorhinus coniceps* without a distinct beak.

***Siphonorhinus larwoodi* (Turk, 1947)**

*Siphonophora larwoodi* Turk 1947: 73, figs 18–20. FEMALE HT (BMNH). India: Uttarakhand, Almora [29.6215441 N, 79.6763696 W]. Lit: **A single female of this species was taken by Capt. H. J. Larwood occuring under stones in a micaceous sand, near the Deodar Hotel, Almora, India, 9.vii.1945*.* Turk 1947: 74.

*Pterozonium larwoodi*–Golovatch and Martens 1996: 167.

*Siphonorhinus larwoodi*–Jeekel 2001: 47.

**Note.** Turk provided a key to six species of Indian *Siphonophora* (1947, pg. 74). Three of these species are now in *Siphonorhinus*: *Siphonorhinus larwoodi*, *Siphonorhinus coniceps*, and *Siphonorhinus cingulatus*.

***Siphonorhinus latus* Silvestri, 1895**

*Siphonorhinus latus* Silvestri 1895: 724. MALE HT (MFS). Indonesia: North Sumatra, Si-Rambé [2.257 N, 99.1114 W]. Lit: **Sumatra: Si-Rambé (E. Modigliani).** Silvestri 1903: 53, fig. 80. Attems 1914: 359. Jeekel 2001: 47.

***Siphonorhinus pallipes* Pocock, 1894**

*Siphonorhinus pallipes* Pocock 1894: 335, pl. 20, fig. 3, 3a. MALE HT (BMNH). Indonesia: West Java, Bogor [-6.6 S, 106.8 W]. Lit: **Java: Buitenzorg; several specimens* (♂♀).* Carl 1912: 508, pl. 9, figs 1–3. Attems 1914: 359. Jeekel 2001: 47.

***Siphonorhinus pellitus* (Attems, 1930)**

*Siphonophora pellita* Attems 1930a: 155, figs 51–61. MALE HT (NMW). Indonesia: Lesser Sunda Islands, Flores Island, Manggarai, Rana Mesé [-8.543 S, 120.713 W]. Lit: **Rana Mesé, West-Flores, 25.6.1927* (♂).* Attems 1930b: 176. Attems 1938: 319. Carl 1944: 260.

*Indiozonium pellitum*–Verhoeff 1941: 215.

*Siphonophorella pellita*–Attems 1951: 254.

*Siphonorhinus pellitus*–Jeekel 2001: 47.

**Note.** Attems (1930a) provided illustrations of the head plus five anterior segments, dorsally (fig. 51) and laterally (fig. 52); vestiture of eighth tergite (fig. 53); eighth pleurite (fig. 54); ventral view of telson, paraprocts, hypoproct (fig. 55); eighth leg tarsus (fig. 56); posterior leg tarsus (fig. 57); left anterior gonopod (fig. 58); left anterior gonopod, closeup of distal portion (fig. 59); left posterior gonopod (fig. 60); left posterior gonopod, closeup of distal portion (fig. 61).

***Siphonorhinus robustus* (Attems, 1938)**

*Teratognathus robustus* Attems 1938: 299, figs 204–218. MALE HT (NMW). Vietnam: Lâm Đồng Province, Di Linh [11.581531 N, 108.076415 W]. Lit: **Djiring (S. Annam), 1.000 m., II.1933*.* Attems 1953: 198.

*Siphonorhinus robustus*–Carl 1941: 573. Jeekel 2001: 47. Likhitrakarn et al. 2014: 475.

**Note.** Attems (1938) provided illustrations of the ventral surface of the gnathochilarium (fig. 204); a magnified view of the left side of the gnathochilarium, including the mentum, stipes and lamella lingualis (fig. 205); dorsal view of the head plus six anteriormost segments (fig. 206); anterior view of the tenth segment in cross-section (fig. 207); ventral surface of the pleurite from segment 12 (fig. 208); coxa and prefemur (fig. 209); and tarsus and claw (fig. 210). Attems (1936) provided three additional localities in addition to the Djiring site: **Dalat, 1.500 m., II.1933** [11.941667 N, 108.438333 W]; **Pic de Lang Biang, 2.400 m.** [12.047222 N,108.44 W], **1.1931; Tayninch (Cochinchina), I.1935** [11.310043 N,106.098275 W].

**Species of uncertain status in *Siphonorhinus***

*Siphonorhinus* sp. Wesener 2014: 417. Madagascar: Antananarivo Province, Ankaratra massif, Manjakatompo Forestry Station [-19.37083 S, 47.339 W]. Lit: **MHNG Mad 89/21; 3* ♂, *2* ♀; *Madagascar, Province Antananarivo, Ankaratra massif, Station Forestière Manjakatompo, près du sommet du Anosirivo, forêt primaire, prèlévement de sol dans une vielle souch, 1980 m; 26.xi.1989, leg. B. Hauser, extraction Berlese à Genève*.*

**Genus *Nematozonium* Verhoeff, 1939**

1 species: South Africa

*Nematozonium* Verhoeff 1939: 216. Type species: *Nematozonium filum* Verhoeff, 1939, by original designation and monotypy. Jeekel 1971: 41. Hoffman 1980: 116. Hamer 1998: 20. Jeekel 2001: 48. Shelley and Hoffman 2004: 218. Enghoff et al. 2015: 386.

***Nematozonium filum* Verhoeff, 1939**

*Nematozonium filum* Verhoeff 1939: 218, pl. 3, fig. 21, pl. 4, figs 21–28. MALE HT (ZSM). South Africa: KwaZulu-Natal Province, Cathkin Peak, Drakensberg escarpment, 1930 m [-29.06724 S, 29.35898 E]. Lit: **In 1930 m. Höhe am Cathkin Peak in den Drakensbergen.** Schubart 1966: 200. Hamer 1998: 20.

*Nematozonium elongatissimum* Verhoeff 1940a: 118. Schubart 1966: 200. Hamer 1998: 20. Shelley and Hoffman 2004: 218 (synonymized).

**Notes.** Verhoeff (1939) provided illustrations of the antenna (pl. 3, fig. 21); ventral surface of the gnathochilarium (pl. 4, fig. 22); lateral surface of the head and collum (pl. 4, fig. 23); mandibles (pl. 4, fig. 24); dorsal surface of the head, collum, and third metatergite (pl. 4, fig. 25); ventral surface of the pleurite and its margin with the tergite (pl. 4, fig. 26); dorsal surface of the telson and terminal ring (pl. 4, fig. 27); and dorsal surface of a paranota and ozopore (pl. 4, fig. 28). Hamer provided the first ever color habitus image of *Nematozonium filum* (Shelley 2015). Other localities for *Nematozonium filum* are as follows (from Shelley and Hoffman 2004): South Africa, Mpumalanga (Graskop, Barberton); KwaZulu-Natal (Bulwer, Drakensberg Mountains, Natal Drakensberg Park/Cathedral Peak, Champagne Castle, Cathkin Peak, Pietermaritzburg, Dlinza Forest Reserve nr. Eshowe).

**Uncertain status in Siphonorhinidae**

Undetermined genus and species Enghoff et al. 1990: 105, fig. 1. Thailand.

Undetermined genus and species Shelley and Golovatch 2011: 125. India: Meghalaya, East Khasi Hills, Cherrapunji [Sohra]. Lit: **Asia: India: Assam: 4 km (4 mi) N Cherrapiniji, 1376 m, 3 October 1961, E.S. Ross, D. Q. Cavagnaro (CASC).**

Undetermined genus and species Wesener 2014: 417. Madagascar: Fianarantsoa Province, Réserve spéciale Ivohibe and Ambalavao National Park; Toliara Province, Rèserve Naturelle Intégrale d’Andohahela.

## Supplementary Material

XML Treatment for Illacme

XML Treatment for Illacme tobini
